# Dose reduction of the new generation biologics (IL-17 and IL-23 inhibitors) in psoriasis: study protocol for an international, pragmatic, multicenter, randomized, controlled, non-inferiority study—the BeNeBio study

**DOI:** 10.1186/s13063-021-05681-z

**Published:** 2021-10-16

**Authors:** Lara S. van der Schoot, Juul M. P. A. van den Reek, Lynda Grine, Lisa Schots, Wietske Kievit, Jo L. W. Lambert, Elke M. G. J. de Jong

**Affiliations:** 1 Radboud University Medical Center, Department of Dermatology, Rene Descartesdreef 1, 6525GL Nijmegen, The Netherlands; 2 Radboud University Medical Center, Radboud Institute for Health Sciences, Nijmegen, The Netherlands; 3 Department of Dermatology, Ghent University Hospital, C. Heymanslaan 10, 9000 Ghent, Belgium; 4 Radboud University Medical Center, Department for Health Evidence, Geert Grooteplein 21, 6525EZ Nijmegen, The Netherlands; 5 Radboud University, Comeniuslaan 4, 6525HP Nijmegen, The Netherlands

**Keywords:** Psoriasis, Dose reduction, Biologics, Non-inferiority, IL-17 inhibitors, IL-23 inhibitors, Therapy

## Abstract

**Background:**

Psoriasis is a chronic immune-mediated inflammatory skin disease for which biologics are effective treatments. Dose reduction (DR) of the first generation biologics seems a promising way for more efficient use of expensive biologics. A substantial part of patients on tumor necrosis factor (TNF)-alfa inhibitors and ustekinumab could successfully lower their dose, after following a tightly controlled DR strategy. The objective of this study is to assess whether controlled DR of interleukin (IL)-17 and IL-23 inhibitors in psoriasis patients with low disease activity is non-inferior (NI) to usual care (UC).

**Methods:**

This is an international, prospective, multicenter, pragmatic, randomized, non-inferiority trial. A total of 244 patients with stable low disease activity (Psoriasis Area and Severity Index (PASI) ≤ 5) for at least 6 months and using secukinumab, ixekizumab, brodalumab, guselkumab, risankizumab, or tildrakizumab in the standard dose, together with stable low disease activity, defined as a PASI ≤ 5 and Dermatology Life Quality Index (DLQI) ≤ 5 at the moment of inclusion, will be randomized 2:1 to DR or UC. In the DR group, dosing intervals will be prolonged stepwise to achieve 66% and 50% of the original dose. Disease activity is monitored every 3 months by PASI and DLQI. In case of disease flare (i.e., PASI and/or DLQI increase), treatment is adjusted to the previous effective dose. The primary outcome is the incidence proportion of persistent flares (PASI > 5 for ≥ 3 months), which will be compared between arms. Secondary outcomes include proportion of patients with successful DR, (course of) PASI and DLQI, serious adverse events (SAEs), health-related quality of life, costs, and pharmacokinetic profile. Outcomes of DR will be compared to UC.

**Discussion:**

With this study, we aim to assess whether DR of IL-17 and IL-23 inhibiting biologics can be achieved for psoriasis patients with low disease activity, without losing disease control. Reducing the dose may lead to more efficient use of biologics.

**Trial registration:**

ClinicalTrials.govNCT04340076. Registered on April 9 2020.

**Supplementary Information:**

The online version contains supplementary material available at 10.1186/s13063-021-05681-z.

## Introduction

### Background

Psoriasis is a chronic, immune-mediated skin disease, which is associated with important comorbidities such as cardiovascular disease and psoriatic arthritis. Disease-related quality of life impairment is large in patients with moderate-to-severe psoriasis [1]. Biologics are effective treatments which have enlarged treatment options for psoriasis patients in the past decades. Biologics block specific cytokines (tumor necrosis factor-alpha (TNFα), interleukin (IL)-12, IL-23, or IL-17) in the psoriasis pathogenesis pathway. The very long-term safety profile is yet to be established. Besides their effectiveness, biologics are expensive and impose a high burden on national healthcare expenditures [2]. Effective and efficient use of biologics is therefore warranted, including the optimal dose for the individual patient.

A new generation of biologics entered the market in recent years: IL-17 inhibitors (IL-17i) (secukinumab, ixekizumab, and brodalumab) and IL-23 inhibitors (IL-23i) (guselkumab, risankizumab, and tildrakizumab). Trial data are promising, with higher effectiveness rates than in first generation biologics like TNFα inhibitors or ustekinumab [3–5]. These biologics are registered in a fixed dose, although not every patient might need this standard dose. Previous research aiming to identify therapeutic windows of biologics showed for example that for adalimumab, one third of patients with good responses had drug levels outside the therapeutic window and were likely to be “overtreated” [6]. Hence, dose reduction (DR) seems a promising method to more efficiently and safely prescribe biologics. In the field of rheumatology, DR of TNFα inhibitors is comparable to continuation of the standard dose in patients with low disease activity [7]. For psoriasis, we recently conducted a randomized non-inferiority trial on DR of adalimumab, etanercept and ustekinumab in patients with stable low disease activity, which showed that in 53% of the patients, the dose was successfully lowered after 12 months [8]. Here, DR was achieved by extending the dosing interval of the biologics. Non-inferiority of DR based on Psoriasis Area and Severity Index (PASI) scores was not demonstrated, yet there was no difference regarding persistent disease flares, defined by disease activity and quality of life measures (PASI and/or Dermatology Life Quality Index (DLQI) scores > 5) during 3 months or longer. Furthermore, the DR strategy resulted in substantial cost savings [9]. Other studies indicated that DR of biologics in psoriasis patients might lead to lower cumulative drug exposure without losing clinical efficacy [10–13]. Direct translation of results from previous DR studies towards the newer biologics cannot be made, due to possible differences between drug classes. To our knowledge, literature regarding DR of IL-17i is sparse, and there are no studies yet that report on DR of IL-23i in psoriasis [14, 15].

With the first generation biologics (TNFα inhibitors), it was shown that discontinuing biologic treatment resulted in quick exacerbations of psoriasis [16]. For IL-17i and IL-23i, withdrawal is also associated with a risk of disease flare, although after retreatment with the original dose a substantial number of patients rapidly regained response [17–22]. Consequently, DR is preferred above treatment withdrawal. To prevent risk of disease flare with DR, we here propose a tightly controlled disease activity-guided strategy.

We designed a multicenter, pragmatic, randomized, controlled non-inferiority study with the aim to identify the number of psoriasis patients that maintain clinical effectiveness and quality of life with a reduced dose of IL-23 and IL-17 inhibiting biologics. We anticipate at least non-inferiority of DR compared to usual care (UC) on the basis of the incidence of persistent disease flares.

## Objectives

The aim of this study is to investigate whether disease activity-guided DR of IL-17 and IL-23 inhibiting biologics for psoriasis patients is non-inferior with regard to persistent disease flares compared to therapy with the standard dose. This translates into the following primary and secondary objectives.

### Primary objective

The primary objective is to assess if tightly controlled DR of IL-17i and IL-23i in psoriasis patients with low disease activity is non-inferior to UC with regard to the incidence proportion of persistent disease flares (PASI > 5 for ≥ 3 months) after 18 months.

### Secondary objectives

1. To assess the proportion of patients with successful DR after 12 and 18 months, defined as using a lower dose than the standard dose and PASI ≤ 5.
2. To assess differences in course of disease activity (PASI) and dermatology-related quality of life (DLQI) in patients with DR versus UC in 18 months.
3. To assess and compare absolute PASI and DLQI scores at month 12 and month 18 in patients with DR versus UC.
4. To assess the incidence of short disease flares (PASI > 5 at one time point) after 18 months in patients with DR versus UC.
5. To assess the time until the first persistent flare (PASI > 5 for ≥ 3 months).
6. To identify predictors for successful DR.
7. To count and compare the number of serious adverse events (SAE) and adverse events of special interest (AEoSI) in patients with DR versus UC. AEoSI include, but are not limited to, infections, malignancies, and joint complaints or new-onset psoriatic arthritis.
8. To assess the pharmacokinetic profile of reduced biologics versus the standard dose.
9. To assess if DR is cost-effective compared to UC.
10. To assess the proportion of patients with initiation of other anti-psoriatic treatments during the study (intensive topical therapies, methotrexate, acitretin) in patients with DR versus UC.

## Methods

### Trial design

This is a multicenter, pragmatic, randomized, controlled non-inferiority trial. This trial will be conducted in 17 medical centers in Belgium and the Netherlands. A comparison will be made between an intervention group (DR) and a control group receiving UC (normal dose). In total, 244 patients will be randomized (2:1). In the intervention arm, the dose of the biologic will be reduced by means of prolongation of the intervals between two doses. We aim to determine whether administration of a reduced dose of the biologic is non-inferior compared to the normal, standard dose. Consequently, a randomized controlled non-inferiority design was chosen.

### Study setting

This study will be carried out in 18 departments of dermatology in Belgium and the Netherlands. Nine centers in the Netherlands will participate: five academic centers (Radboud University Medical Center Nijmegen, Erasmus Medical Center Rotterdam, Maastricht University Medical Center, University Medical Center Utrecht, University Medical Center Groningen) and five non-academic centers (Bravis hospital Bergen op Zoom, Catharina hospital Eindhoven, Gelre hospital Apeldoorn, Ziekenhuisgroep Twente Almelo/Hengelo, and Slingeland hospital Doetinchem). In Belgium, the participating centers include five academic centers (Ghent University Hospital, Cliniques Universitaires Saint-Luc Brussels, Centre Hospitalier Universitaire (CHU) de Liège, Erasme Hospital Brussels, University Hospital Leuven) and three non-academic centers or private practices (AZ Maria Middelares Ghent, AZ Sint-Lucas Ghent, and Dermatologie Maldegem).

Ethical approval for this study was obtained from the Medical Ethical Committee (Arnhem-Nijmegen) for the Dutch sites and from the competent authorities (FAMHP) and the Ethics Committee of University Hospital Ghent and University Ghent after consulting the Ethics Committees of each participating site in Belgium. Written informed consent will be obtained from each participant.

### Eligibility

Adult patients with plaque psoriasis who are treated with IL-17i or IL-23i in the standard, registered dose for at least 6 months, and who have stable low disease activity, are eligible. Low disease activity is defined as PASI ≤ 5 in the previous 6 months and at the moment of inclusion, together with a DLQI ≤ 5 at inclusion. In case no PASI scores are available, it should be clear from the patient record that the psoriasis was “clear” or “almost clear” in the past 6 months. A PASI ≤ 5 is chosen based on experts’ opinion and our previous DR study [8]. The DLQI score was added in order to adjust to the impact of psoriasis on the patients’ quality of life [23]. A DLQI ≤ 5 indicates mild influence on quality of life [24]. In order to establish whether good disease control is present, a combination of PASI as a clinical outcome measure and DLQI as a patient reported outcome measure will be assessed every 3 months during the study (tight control). Patients who are eligible for inclusion in this study must meet the following criteria:

### Inclusion criteria

- Plaque psoriasis (primary indication for biologic).
- Treatment for at least 6 months with IL-23i or IL-17i in the standard dose (dose advised by the label).
- PASI ≤ 5 in the previous 6 months. If no PASI scores are available in the previous 6 months, it should be clear from the patient record that the psoriasis was clear or almost clear.
- PASI ≤ 5 at inclusion.
- DLQI ≤ 5 at inclusion.

### Exclusion criteria

- Another indication than plaque psoriasis as the main indication for biologic use (e.g., psoriatic arthritis).
- Concomitant use of systemic immunosuppressants other than methotrexate or acitretin.
- Severe comorbidities with short life-expectancy.
- Presumed inability to follow the study protocol.

### Recruitment

All patients who are eligible for this study will be asked by their treating physician. They will receive oral and written information from the local investigator. The investigator will obtain written informed consent and the patient will be randomized. The dosing schedule will be explained depending on which biologic the patient uses. Patients can leave the study any time for any reason without consequences. The investigator can decide to withdraw a subject from the study for urgent medical reasons. When subjects are withdrawn from the study, they will not be replaced.

### Randomization, blinding, and treatment allocation

The investigator will enroll participants. After including the participant and obtaining written informed consent, the investigator will enter participants in a web-based randomization program (Castor). Participants will be allocated to each group (DR or UC) by this web-based randomization program (Castor) that generates block randomization (variable block size of 6, 9, 12) with a random (2:1) allocation sequence and stratified by biologic. After randomization, the randomization group is visible for the investigator and the investigator will inform the participant. Patients will be randomized 2:1 to DR or continuation of the normal dose (UC). The ratio of 2:1 is chosen to be able to include more determinants in an analysis for successful DR. Due to the pragmatic character of this trial, and due to the nature of the intervention (injections), patients and investigators will not be blinded. Patients in the DR group will receive secukinumab, ixekizumab, brodalumab, guselkumab, risankizumab, or tildrakizumab, and doses will be lowered according to the schedule as described below (Table 1). Patients in the control group will receive the normal, standard dose of secukinumab, ixekizumab, brodalumab, guselkumab, risankizumab, or tildrakizumab without interval prolongation.

**Table 1:** Dose reduction (DR) steps per biologic

Biologic	Normal dose	First step DR	Second step DR
Secukinumab	300 mg/4 weeks	300 mg/6 weeks	300 mg/8 weeks
Ixekizumab	80 mg/4 weeks	80 mg/6 weeks	80 mg/8 weeks
Brodalumab	210 mg/2 weeks	210 mg/3 weeks	210 mg/4 weeks
Guselkumab	100 mg/8 weeks	100 mg/12 weeks	100 mg/16 weeks
Risankizumab	150 mg/12 weeks	150 mg/18 weeks	150 mg/24 weeks
Tildrakizumab	100 or 200 mg/12 weeks	100 or 200 mg/18 weeks	100 or 200 mg/24 weeks

*Abbreviations*: *DR* dose reduction, *mg* milligram

### Study groups

### Control group

Patients in the control group will continue treatment with the normal, standard dose based on the prevailing national guidelines. Treatment decisions are made at the discretion of the treating physician following these guidelines. Control visits are planned every 3 months and patients are explained to contact their physician when they experience increased disease activity. The PASI and DLQI are performed during 3-monthly outpatient clinic visits. Topical therapies and concomitant use of methotrexate or acitretin are allowed. In case of a disease flare, treatment will be adjusted. Topical and/or systemic therapy will be optimized and when required the dose of the biologic will be increased or the biologic will be switched to another agent. In case of treatment alternations, the patient will remain in the study for follow-up.

### Dose reduction group

**Fig. 1:**
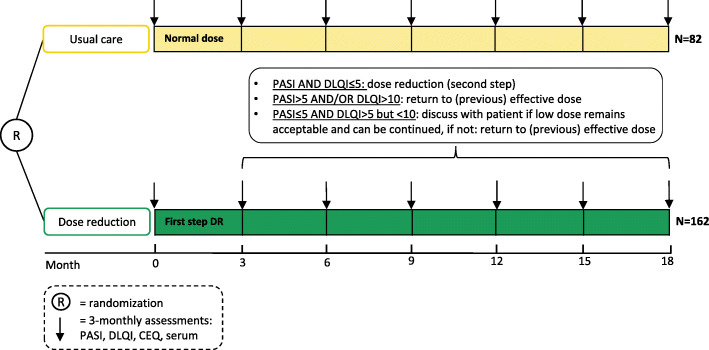
Participant timeline. Patients (*n* = 244) using secukinumab, ixekizumab, brodalumab, guselkumab, risankizumab, or tildrakizumab will be randomized to dose reduction or usual care. Control visits will be scheduled every 3 months for assessment of PASI, DLQI, cost-effectiveness questionnaires (EQ-5D-5L, SF-36, iMTA MCQ, and PCQ), drug levels, and anti-drug antibodies. CEQ, Cost-Effectiveness Questionnaires; PASI, Psoriasis Area and Severity Index; DLQI, Dermatology Life Quality Index

The doses of secukinumab, ixekizumab, brodalumab, guselkumab, risankizumab, or tildrakizumab will be lowered by interval prolongation in two steps to 66% and 50% of the original dose, respectively (Table 1). The intervals of drug administration will be prolonged depending on the PASI and DLQI score (Fig. 1). DR is allowed in case of low disease activity, defined as PASI ≤ 5 and DLQI ≤ 5. First, the dose will be decreased to 66% of the normal dose of the biologic (by interval prolongation with a factor 1.5). After 3 months, if there remains low disease activity (PASI ≤ 5 and DLQI ≤ 5), the dose will be further reduced to 50% of the original dose (by doubling the original interval). When disease flare occurs (PASI > 5 and/or DLQI > 10), or when the patient is not willing to further use the lower dose, the patient will return to the previous effective dosing interval. In case of PASI ≤ 5 and DLQI > 5 but ≤ 10, it will be discussed with the patient if the reduced dose remains acceptable and can be continued or not. If possible, the patient will stay on the lowest dose. Each step will be analyzed after 3 months, or when the patient visits earlier due to complaints. In case a patient returned to the previous effective dosing interval, the dose will not be reduced again at a later time point. Topical therapies and concomitant use of methotrexate or acitretin are allowed. When there is still no disease control after reintroduction of the normal dose, treatment will be adjusted. Topical and/or systemic therapy will be optimized and, when required, the dose of the biologic will be increased or patients will switch to another agent. In case of treatment alternations, the patient will remain in the study for follow-up.

### Procedures and outcome measures

At baseline, patient and treatment characteristics will be collected, such as sex, age, treatment history, comorbidities, and disease duration. Patients will be followed for 18 months and regular visits will be planned at baseline, month 3, 6, 9, 12, 15, and 18. An overview of interventions and assessments by study time point is presented in Table 2. If a patient has a disease flare outside the regular visits, an additional study visit will be planned. Every 3-monthly visit, PASI and DLQI scores will be retrieved, and blood samples will be drawn for determining anti-drug antibody levels and modeling drug trough levels. Also, dosage schedules, adverse events, and concomitant topical and systemic medication use will be registered. Medication dispensing records will be collected for each participant from their pharmacies. Patients will be asked to fill in patient diaries with information on medication use. Questionnaires relevant for cost-effectiveness analysis will be completed in by the patients at these visits. These include the European Quality of Life-5 Dimensions-5 Level (EQ-5D-5L) questionnaire [25], the Short Form (SF)-36 questionnaire [26], and adapted versions of the “institute for Medical Technology Assessment” (iMTA) Medical Consumption Questionnaire (MCQ) and Productivity Cost Questionnaire (PCQ) [27].

**Table 2:**
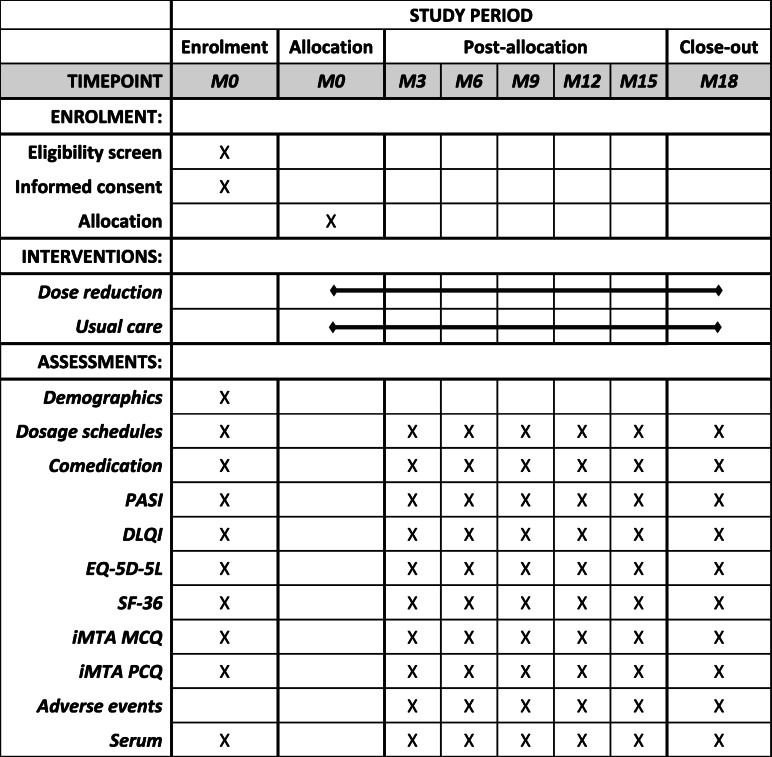
Schedule of enrolment, interventions, and assessments by study time point

*Abbreviations*: *M* months, *PASI* Psoriasis Area and Severity Index, *DLQI* Dermatology Life Quality Index, *EQ-5D-5L* European Quality of Life-5 Dimensions-5 Level questionnaire, *SF-36* Short Form-36 questionnaire, *iMTA MCQ* institute for Medical Technology Assessment Medical Consumption Questionnaire, *PCQ* Productivity Cost Questionnaire

Our primary outcome is non-inferiority of the incidence proportion of persistent flares (PASI > 5 for ≥ 3 months) in the intervention group. If the 95% confidence interval from the difference in incidence of persistent flares in the intervention group versus control group exceeds the non-inferiority margin (15% difference in persistent flares) at 18 months of follow-up, it can be concluded that non-inferiority could not be demonstrated for DR.

Our secondary study outcomes are as follows:

- Whether participants will have successful DR after 12 and 18 months, defined as using a lower dose than the normal dose and PASI ≤ 5.
- Psoriasis disease activity, measured with the Psoriasis Area and Severity Index (PASI) at each 3-monthly study visit. The PASI is a composite measure of erythema, scaling, induration, and extensiveness of the psoriasis plaques [28]. It results in a single score for psoriasis severity ranging from 0 to 72 with lower scores indicating lower disease activity.
- Dermatology-related quality of life as measured with the Dermatology Life Quality Index (DLQI) at each 3-montly study visit. The DLQI is a practical questionnaire for routine clinical use and consists of 10 questions surveying the impact of skin disease on health-related quality of life. A total sum score ranging from 0 to 30 is derived from all questions, with lower scores indicating limited impact on disease-related quality of life [23].
- Whether participants will have short disease flares throughout the study period (18 months), defined as a PASI > 5 at one time point.
- Whether other anti-psoriatic medication will be initiated in participants during the study period (18 months). Investigators will report start of other medications at every study visit.
- Whether participants will have serious adverse events (SAE) and adverse events of special interest (AEoSI) during the study period. AEoSI include, but are not limited to, infections, malignancies, and joint complaints or new-onset psoriatic arthritis. All adverse events reported spontaneously by the participant or observed by the investigator will be recorded in the CRF during the study period (18 months).
- Drug trough levels of each included drug will be measured in blood serum samples which will be collected from participants at each 3-montly time point. Serum samples will be analyzed through an enzyme-linked immunosorbent assay (ELISA) for serum drug and anti-drug antibody levels.
- Anti-drug antibody levels of each included drug will be measured in blood serum samples which will be collected from participants at each 3-montly time point. Serum samples will be analyzed through an enzyme-linked immunosorbent assay (ELISA) for serum drug and anti-drug antibody levels.
- Utilities will be derived from EuroQoL 5 Dimensions (EQ-5D-5L) questionnaires, which will be measured at each 3-montly time point. The EQ-5D-5L questionnaire consists of 5 dimensions (mobility, self-care, usual activities, pain/discomfort and anxiety/depression), and each dimension has 5 levels: no problems, slight problems, moderate problems, severe problems, and extreme problems. In addition, patients are asked to complete a visual analog scale to rate their health status at a scale of 0–100 [25]. Utility scores will be used to calculate quality-adjusted life years (QALYs) which are used to determine cost-effectiveness of DR.
- Health status of participants will be assessed by using the Short Form 36 (SF-36) version 2 questionnaire at every 3-monthly time point. The SF-36 consists of 36 questions regarding 8 domains of health, including physical functioning, physical role, pain, general health, vitality, social function, emotional role, and mental health [26, 29]. Scores from all domains will be summarized with differing weightings to calculate mental component scores (MCS) and physical component scores (PCS). Utilities will be derived as well from the SF-36 for sensitivity analyses, calculated based on the 12 specific SF-36 questionnaire answers included in the SF6D system.
- Volumes of care, as measured with the iMTA Medical Consumption Questionnaire (MCQ) at each 3-monthly time point. The iMTA MCQ measures all relevant health care related costs like outpatient visits at any medical specialist, hospitalizations, and imaging procedures [27]. Scores will be used to calculate direct medicals costs and non-medical costs.
- Loss of productivity and presenteeism of participants, as measured with the iMTA Productivity Cost Questionnaire (PCQ) at each 3-monthly time point. The iMTA PCQ measures patient-reported absences from paid or unpaid labor [30]. Scores will be used to calculate direct medicals costs and non-medical costs.

### Definition of disease flare

We defined a psoriasis flare as a PASI score > 5. A short disease flare was defined as a flare at one time point, and a persistent flare as a PASI > 5 for at least 3 months. Successful DR was defined as use of a lower biologic dose and a PASI ≤ 5. A PASI cutoff of 5 was chosen in absence of a validated definition for psoriasis flare. This cutoff was based on our previous DR trial [31], where PASI 5 was chosen based on expert opinion and on previous data that showed that patients who remain on a biologic reach average PASI scores ≤ 5 [32]. Although disease flare is based on disease activity only, quality of life measured by DLQI is incorporated in the tightly controlled DR strategy. In patients where PASI 5 reflects active disease, patients can always return to their previous effective dose or the normal dose when they experience larger impact of their psoriasis on their quality of life. Incidence of persistent flares was chosen as primary outcome to assess non-inferiority. Brief, temporary flares that improve after treatment adjustments or reintroduction of higher doses, and without large impact on overall disease control, might occur more frequent in a DR arm and are inherent to such a strategy. However, to not harm the patient, such effects should not last too long. For this reason, persistent flares are chosen as the primary outcome. PASI is used instead of Psoriasis Global Assessment (PGA) as PASI is a common used tool in Europe daily practice and in clinical trials. Assessment of PASI is standardized, whereas for PGA different scales and subtypes are used [33]. PASI has been reported to be a reliable instrument to evaluate treatment success when measured at baseline and during treatment [34].

### Power and sample size analyses

The primary outcome is non-inferiority of persistent flares (PASI > 5 for ≥ 3 months) in the intervention group with a non-inferiority margin of 15%. If the upper limit of the 95% confidence interval of the difference in persistent flares between the intervention group versus control group exceeds 15% at 18 months of follow-up, it must be concluded that non-inferiority could not be demonstrated for DR [36]. A margin of 15% was chosen based on clinical grounds, and on previous DR studies [8, 37]. Inherent to the intervention, there could be a small increase in disease flares (i.e., loss of disease control) as drug dosages are lowered. However, it is expected, based on other studies [8, 35], that most flares will be easy to control in the end without residual damage for patients, for example by reintroduction of a higher dose or switch to another biologic. It is therefore expected that persistent flares will not occur very frequently, as has also been shown in the CONDOR trial [8]. In addition, in the UC arm, there will be disease flares as well, as PASI scores might fluctuate over time and some patients might experience loss of effectiveness of their biologic [38]. The chosen non-inferiority margin of 15% is to some extent arbitrary, but refers to a clinically acceptable difference in persistent flares. Of note, the point estimate should be much lower than the margin of 15%, because the margin refers to the upper limit of the 95% confidence interval of the difference in persistent flares which should not exceed 15%. Therefore, we found a margin of 15% (hence a point estimate << 15%) for the difference in persistent flares acceptable, especially because we know that effectiveness could often be regained in patients with persistent flares in studies on other biologics [8, 35]. With an expected chance of being able to reduce biologic dosages in > 50% of patients [8], the benefit-harm ratio seems well balanced by accepting this anticipated increase of persistent flares in patients undergoing DR compared to UC.

With this margin, one-sided testing (*α* = 0.025; 1 − β = 0.8) and a randomization ratio of 2:1 DR versus UC, we calculated that 222 patients need to be included to reject the null-hypothesis of inferiority. Taking a drop-out rate of 10% into account, 244 patients need to be included, with 162 in the DR arm and 82 in the UC arm.

### Data collection and management

All data will be collected and entered in Castor, an electronic data management system, which is setup for clinical trials [39]. Data will be coded and kept based on the rules for good clinical practice (GCP) by GCP-certified personnel [40]. Handling of personal data will comply with the General Data Protection Regulation [41]. All blood samples will be coded before sending to Ghent University Hospital for storage and to University Hospitals Leuven where the samples will be analyzed.

### Statistical analyses

All analysis will be done according to a per protocol analysis, as this is the preferred and most conservative analysis for non-inferiority studies [42]. Of note, all patients that follow the DR protocol (Fig. 1) will remain in the group they were allocated to, including DR patients that returned to their normal dose according to the protocol. Patients on a lower dose who should return to a higher dose according to the protocol are not included in per protocol analysis after the moment of protocol deviation. They will however complete the study procedures, and occurred protocol deviations will be summarized and reported. Subjects lost to follow-up will be included in analyses until their lost to follow-up date only. Information about patients lost to follow-up will be described. Intention-to-treat (ITT) analysis will be performed as well on all outcomes for sensitivity reasons, and as they are needed for the cost-effectiveness analysis. Data of patients who deviated from the protocol will be included in ITT analysis. For the ITT analyses, last observation carried forward (LOCF) will be used for imputation of missing data. The last available data from patients who are lost to follow-up will be imputed. Extent and nature of missing data will be described. Patient and treatment characteristics will be summarized as means or medians and percentages, depending on the type of measurement.

The primary outcome incidence proportion of persistent flares will be calculated for both groups. Number of patients with a persistent flare will be presented as proportions with corresponding confidence intervals. Confidence intervals of proportions will be calculated by Fishers exact tests (Clopper-Pearson), and the proportions will be compared using Fisher exact tests (open source calculator OpenEpi, V3.01) [43]. If the difference in incidence proportion of persistent flares in the intervention group versus the UC group exceeds the non-inferiority margin (15% difference in persistent flares) at 18 months of follow-up, it will be concluded that non-inferiority could not be demonstrated for DR. Time until the first persistent flare in both groups will be graphically presented by Kaplan-Meier survival estimation.

The proportion of patients with successful DR will be expressed using descriptive statistics. PASI and DLQI course throughout the study will be compared between the intervention and control groups using mixed methods analysis. All disease-activity scores (PASI) will also be directly compared at each time point (every 3 months) between the two groups using an unpaired *t* test or a non-parametric alternative. PASI and DLQI at 12 and 18 months will be analyzed with ANCOVA in which the baseline values will be included as a covariate to gain efficiency. A multivariable regression analysis will be carried out in order to identify predictors for successful DR at month 12 and month 18. Possible candidate predictors will include baseline patient and treatment characteristics and baseline trough drug concentrations. Based on group size, we will test the four most promising variables. Proportions, rate ratios, and relative rate ratios, with corresponding confidence intervals, of SAEs and AEoSI will be described and differences between groups will be tested using classical statistical methods (Fisher exact test). SAEs and AEoSI *related to* DR will also be counted and expressed as proportions and rate ratios. A subanalysis for all outcomes will be made for the different drug classes and on individual drug level if appropriate (depending on numbers included per class).

### Cost-effectiveness analysis

Cost-effectiveness of DR will be calculated based on health-status (SF-36), utilities measured with EQ-5D-5L, volumes of care (iMTA Medical Consumption Questionnaire), and loss of productivity and presenteeism (iMTA Productivity Cost Questionnaire). Utilities will be estimated by weighing the scored answers on the EQ-5D-5L with the local tariffs and using the trapezium rule quality-adjusted life years (QALYs) will be calculated. A sensitivity analyses will be conducted by calculating QALYs based on utilities from the SF6D system, derived from answers on the 12 specific SF-36 questionnaire answers. Cost prices for each volume of consumption will be determined based on standard local cost prices. Productivity losses will be valued by means of the friction cost method. Volumes of care will be multiplied with the cost prices for each volume of care to calculate costs. Because we anticipate non-inferiority of the DR strategy, cost-savings will be analyzed. Direct medical cost as well as total costs (medical and non-medical costs) will be compared between the intervention and control group. A possible small but acceptable loss of effect can be incorporated in the analyses by determining a decremental cost-effectiveness ratio (DCER) by dividing the difference in costs by the difference in quality-adjusted life-years (QALYs) between the groups. The DCER expresses with how much money a loss of 1 QALY is compensated. If this amount is high, decision-makers are willing to accept a loss of effect. Uncertainty in the DCER will be non-parametrically determined using bootstrap techniques (1000 replications). Results from this analysis will be presented in a scatter plot and willingness to pay (or accept) curve. Furthermore, the net monetary benefit (NMB) per patient will be calculated for different levels of willingness to pay (WTP) in dollars per QALY, using the formula: WTP * effect (difference in QALY) − costs. This results in the net amount of money saved, when the possible loss of QALY is corrected for, using different WTP levels per QALY.

### Pharmacokinetic analyses

Pharmacokinetic analyses will be performed for each biologic to determine anti-drug antibody levels and drug trough levels. Modeling of drug and anti-drug antibody levels will be done based on Bayesian statistics with NONMEM to gain insight in the clearance of the biologics during DR and to identify factors that influence pharmacokinetics. A maximum of variables (age, gender, disease severity, disease duration, dosing scheme (day of injection), etc.) will be introduced in the model where possible. The validity of the model will be assessed through goodness of fit with the aim to model the area under curve estimation.

### Oversight and monitoring

The study will be overseen by the trial steering committee (TSC). The TSC consists of the research committee, funders, statistician, and patient representatives. The research committee consists of the principal and coordinating investigators of the sponsor (Radboud University Medical Center) and national coordinating center (Ghent University Hospital) and an independent expert. The responsible ethical committees and competent authorities require annual reports. No other audits will be performed, unless requested by the study sponsor, funding source, or the responsible competent authorities.

Data of all centers will be monitored following guidelines of the Radboud University Medical Center for the Dutch sites and the guidelines of Ghent University Hospital for the Belgian sites. A data safety monitoring board (DSMB) will not be installed as this study is judged as a negligible risk trial.

All adverse events will be recorded in the source document (patient medical record) and electronic data management system after randomization. Safety reviews will be performed annually by the TSC. Interim safety reviews will be performed when this is deemed necessary. The sponsor will suspend the study if there is sufficient ground that continuation of the study will jeopardize subject health or safety. The sponsor and national coordinating center have a full insurance that covers the costs of potential harms.

All major protocol modifications will be approved by the responsible ethical committees, and participants will be reconsented as necessary. Changes will be added to the ClinicalTrials.gov protocol. Upon trial finalization, findings will be submitted for publication to an open-access peer-reviewed journal. Results will be presented at relevant national and international conferences, as well as in relevant patient associations. All publications will be in accordance with international recognized scientific and ethical standards concerning publications and authorship, including the Uniform Requirements for Manuscripts Submitted to Biomedical Journals, established by the International Committee of Medical Journal Editors. There is no intended use of professional writers.

## Discussion

DR of biologics in patients with low disease activity seems a promising way to provide personalized treatment, improve safety, and reduce healthcare costs. Until now, DR has mainly been described for TNFα inhibitors and ustekinumab [10–13]. The possibility of DR is only mentioned in a few guidelines [44, 45]. To our knowledge, the current study is the first randomized, controlled trial designed to investigate disease activity-guided DR of IL-17i and IL-23i for psoriasis patients with low disease activity in a multicentric and pragmatic setting.

This study was partially based on our previous tightly controlled DR study on adalimumab, etanercept, and ustekinumab [8]. We chose a different primary outcome, i.e., incidence of persistent flares, instead of difference in disease activity. Disease activity should certainly be incorporated in the primary outcome, as it is the domain that should be non-inferior. Because of the tightly controlled strategy however, disease activity could possibly not differ between the two groups at study end. Therefore, disease activity should be analyzed over time, but time-integrated disease activity measures are more difficult to interpret and less informative for daily practice than percentage of patients with a flare. Incidence of flares is therefore chosen as primary outcome in the majority of DR studies [37]. Persistent flares are clinically more important than short disease flares due to larger impact on overall disease control. For this reason, persistent flares are chosen as the primary outcome. In addition, inclusion criteria are less strict in the current study compared with the previous study, as patients can also be included in case no PASI scores were available in the past 6 months. We believe that this might improve external validity and practicability, as PASI is not measured in every clinic. We extended the follow-up duration compared to the previous study to 18 months instead of 12 months, because biologics with relatively long dosing intervals (risankizumab and tildrakizumab) were included. As the DR schedules of these biologics might extend the 3-monthly visits in time, there should be more time to assess the DR effect. A longer follow-up duration allows longer term safety analysis as well.

The strength of this study is that it will be performed in two different countries, and in various academic and non-academic centers, to improve external validity. Moreover, the real-world practice setting, flexibility of treatment schedule, and outcomes relevant to patients lead to a highly pragmatic trial of which outcomes have a high generalizability [46]. A possible limitation is the open-label design of this study, as reporting bias might occur for patient reported outcomes such as adverse events. However, as said, the aim of this pragmatic study is to provide high external validity, which would be lowered by blinding.

The focus of this study is on the strategy of DR in general. Hence, the study is powered for the total group and not per biologic. In addition, subanalyses per drug class and per biologic will be performed when appropriate.

More knowledge on DR of biologics might contribute to more efficient and effective use of biologics. The COVID19 situation has emphasized the need for more research regarding personalized dosing and the possibility of lower dosages of immunomodulatory biologics [47]. In this prospective, multicenter, randomized controlled non-inferiority trial, the possibility of DR of the newer generation biologics (IL-17i and IL-23i) will be investigated. If DR is non-inferior to UC with standard dosages, we can provide patients with more personalized treatment. This may lead to lower cumulative doses of therapy, a lower risk of side-effects, and reduction of healthcare costs.

## Trial status

Recruitment started at the 20 August 2020. Last visit is planned for July 2023. Current protocol version 1.5, date 29 July 2021.

## Supplementary Information

**Additional file 1:** Appendix 1. Model informed consent forms BeNeBio study.

## Data Availability

After the trial, data will be maintained and will be kept in a dedicated and secure workspace (Radboudumc Digital Research Environment). We plan to share anonymized study data in a data repository. Pseudonymized data can be shared with public health institutions in member states of the European Union that help to substantiate the decision on reimbursement of medical treatments. These government institutions can only analyze study data and use it for the treatments that are part of this study whether or not to reimburse in their country. Participants should give their permission for sharing their data within the written informed consent form.

## Abbreviations

AEoSI: Adverse events of special interest
DCER: Decremental cost-effectiveness ratio
DLQI: Dermatology Life Quality Index
DR: Dose reduction
DSMB: Data Safety Monitoring Board
EQ-5D-5L: European Quality of Life-5 Dimensions-5 Level
IL: Interleukin
ILi: Interleukin inhibitor
iMTA: Institute for Medical Technology Assessment
ITT: Intention to treat
LOCF: Last observation carried forward
MCQ: Medical Consumption Questionnaire
NI: Non-inferior
NMB: Net monetary benefit
PASI: Psoriasis Area and Severity Index
PCQ: Productivity Cost Questionnaire
PGA: Physician Global Assessment
QALY: Quality-adjusted life-years
SAE: Serious adverse events
SF-36: Short Form-36
TNF: Tumor necrosis factor
TSC: Trial Steering Committee
UC: Usual care
WTP: Willingness to pay
