# Supplementary material for: Dose reduction of the new generation biologics (IL-17 and IL-23 inhibitors) in psoriasis: study protocol for an international, pragmatic, multicenter, randomized, controlled, non-inferiority study—the BeNeBio study
Source: Trials. 2021 Oct 16;22:707. doi: 10.1186/s13063-021-05681-z (PMC8520290; doi:10.1186/s13063-021-05681-z)
Supplement: Supplementary file 1 — Additional file 1: Appendix 1. Model informed consent forms BeNeBio study. [file 13063_2021_5681_MOESM1_ESM.pdf]

PATIENTENINFORMATIE: 'Het afbouwen van biologics bij psoriasis patiënten met lage ziekte-activiteit'

Geachte heer/mevrouw,

Wij vragen u vriendelijk om mee te doen aan een medisch-wetenschappelijk onderzoek. U beslist zelf of u wilt meedoen. Om mee te doen is uw schriftelijke toestemming nodig. Voordat u de beslissing neemt, is het belangrijk om meer te weten over het onderzoek. Lees deze informatiebrief rustig door. Bespreek het met partner, vrienden of familie. Hebt u na het lezen nog vragen, dan kunt u terecht bij de onderzoeker. Ook kunt u terecht bij een onafhankelijk persoon, die veel weet van het onderzoek. U vindt de namen en telefoonnummers bij de contactgegevens aan het einde van deze brief.
Verdere informatie over meedoen aan wetenschappelijk onderzoek in het algemeen kunt u vinden in de (online) brochure 'Medisch-wetenschappelijk onderzoek'.
(https://www.rijksoverheid.nl/documenten/brochures/2014/09/01/medisch-wetenschappelijk-onderzoek-algemene-informatie-voor-de-proefpersoon)

Waarom dit onderzoek?

De afdelingen Dermatologie van het Radboudumc Nijmegen (Nederland), Universitair Ziekenhuis Gent (België) en omliggende streekziekenhuizen doen onderzoek naar de behandeling van psoriasis. U gebruikt al enige tijd een biologic (secukinumab (Cosentyx®), ixekizumab (Taltz®), brodalumab (Kyntheum®), guselkumab (Tremfya®), risankizumab (Skyrizi®), tildrakizumab (Ilumetri®)) en uw huid reageert hier goed op. Omdat er over andere biologics aanwijzingen zijn dat een lagere dosis net zo effectief kan zijn, willen wij nagaan of dit bij deze middelen ook zo is. Dit kan ertoe leiden dat sommige mensen langdurig met een lagere dosis kunnen worden behandeld terwijl de psoriasis rustig blijft. Wij willen onderzoeken of dit mogelijk tot minder bijwerkingen (op de lange termijn) leidt en of de kosten dalen. Omdat we niet willen dat de ziekte onnodig erger wordt, willen wij dit onderzoeken op een manier waarin we patiënten goed kunnen begeleiden. Mensen die niet goed reageren op de lagere dosis, kunnen weer terug naar hun normale dosering.

Wat vragen wij van u, en wat is er anders dan de huidige behandeling die u krijgt?

Wij willen u graag elke 3 maanden op de polikliniek controleren gedurende 1.5 jaar in het kader van deze studie. Er worden twee groepen gemaakt, en door loting zal bepaald worden in welke groep u komt.

Er zijn 2 mogelijkheden: het kan zijn dat u in de zogenaamde **'controle groep'** terecht komt. U kunt dan uw huidige behandeling gewoon doorzetten. Wel vragen wij u gedurende de studie tijdens elke visite online vragenlijsten in te vullen en 1 extra buisje bloed af te staan zoveel mogelijk op een moment dat u al geprikt wordt vanwege uw behandeling. Zoals u wellicht gewend bent beoordelen wij de ernst van de psoriasis tijdens elke visite. Er wordt een persoonlijk e-mailadres van u gevraagd waar een link naar de vragenlijsten naartoe wordt verstuurd. De vragenlijsten worden door u in een beveiligde omgeving ingevuld.

Als u in de **'dosis-afbouw groep'** geloot wordt, dan zal de dosis langzaam afgebouwd worden

voor zover de ziekteactiviteit en uw kwaliteit van leven dit toelaten. We zullen maximaal tot de halve dosering van uw huidige behandeling afbouwen (als u bijvoorbeeld guselkumab (Tremfya®) 1 spuit per 8 weken gebruikt, zullen we het maximaal verlagen naar guselkumab 1 spuit per 16 weken). Als de psoriasis teveel toeneemt of uw kwaliteit van leven score slechter wordt, zal de oude dosering in overleg met u hervat worden. Wij vragen u bij elke visite online vragenlijsten in te vullen en 1 extra buisje bloed af te staan, zoveel mogelijk op een moment dat u al geprikt wordt vanwege uw behandeling. Zoals u wellicht gewend bent scoren wij de ernst van de psoriasis tijdens elke visite. De visites kunnen iets langer duren, met name tijdens de eerst visite, waarin we alles omtrent de studie uitleggen. Er wordt een persoonlijk e-mailadres van u gevraagd waar de vragenlijsten naartoe worden verstuurd. De vragenlijsten worden door u in een beveiligde omgeving ingevuld.

Uw behandelaar zal soms ook de onderzoeker van de studie zijn. Mocht u een andere behandelaar wensen die niet bij de studie betrokken is, dan kunt u dit aangeven.

Wat gebeurt er als u niet wilt deelnemen aan dit onderzoek?
Wij benadrukken dat uw deelname volledig vrijwillig is. Met andere woorden: u hoeft niet deel te nemen als u dit niet wilt en er verandert dan niets aan uw huidige behandeling. Daarnaast kunt u stoppen met de studie op ieder moment dat u dat wenst. Als u wenst deel te nemen aan dit onderzoek verzoeken wij u een schriftelijke toestemming te ondertekenen. Dit betekent dat u deze informatie heeft gelezen en begrepen. Tevens geeft u toestemming aan onderzoekers, aangesloten bij dit onderzoek, om in uw dossier gegevens t.a.v. de studie op te zoeken. De studiegegevens zullen gepseudonimiseerd (wel ontdaan van naam, geboortedatum en adresgegevens) worden vastgelegd in een digitaal gegevensbestand.

Wat zijn de voor- en nadelen van deelname voor u, en welke bijwerkingen kunt u verwachten?
U hebt 67% kans dat u in de groep terecht komt waarin u uw medicatie het komende jaar gaat afbouwen. Als uw psoriasis rustig blijft, en u er een goede kwaliteit van leven bij behoudt, zal u op de halve dosering uitkomen van wat u nu gebruikt. Concreet dient u uzelf dus minder injecties toe. Mogelijk zien we dat u minder kans op (lange termijn) bijwerkingen krijgt, dit weten we nog niet en moet onderzocht worden. Soms komt het voor dat de psoriasis toeneemt. We zullen dan de oude dosering hervatten of u zo nodig behandelen met extra medicatie. **U kunt tussen de visites door contact met ons opnemen via de polikliniek zodat u niet hoeft te wachten tot u weer een afspraak heeft.** U hoeft daarna niet opnieuw af te bouwen maar we blijven u wel op de polikliniek controleren en vragenlijsten afnemen gedurende de studie.

Patiënten die ook **'artritis psoriatica'** hebben, gewrichtsontstekingen passend bij psoriasis, kunnen mogelijk een toename van de gewrichtsklachten krijgen bij het minderen van de dosis. Wij willen u vragen contact met uw behandelend dermatoloog en reumatoloog op te nemen wanneer dit het geval is. Wij zullen vooraf aan dosis afbouwen ook met uw reumatoloog overleggen of hij/zij hiermee akkoord gaat.

Mogelijk is er een kleine kans dat u antistoffen tegen de biologic gaat vormen en dat hierdoor de biologic minder goed gaat werken. Wij denken dat deze kans zeer klein of zelfs afwezig is. Wel willen we dit graag onderzoeken en daarom prikken we extra bloed. Als u niet afbouwt, hebt u geen direct voordeel van dit onderzoek. Wel zult u in toekomst van de opgedane kennis kunnen profiteren omdat wij de resultaten in de praktijk willen toepassen.

Het onderzoek kost u elke 3 maanden tijd (ongeveer 20 minuten) om de vragenlijsten in te vullen, ongeacht in welke behandelgroep u terecht komt. Extra bloedbepalingen worden zoveel mogelijk tijdens uw normale bloedprikafspraken meegenomen, dus u hoeft in principe niet vaker geprikt te worden tenzij u normaliter niet elke 3 maanden geprikt werd. Er wordt 1 extra buisje bloed afgenomen van 10ml.

Hoe is de privacy geregeld bij dit onderzoek en wat gebeurt er met het lichaamsmateriaal?
Mensen die uw gegevens kunnen inzien zijn het onderzoeksteam, de veiligheidscommissie die het onderzoek in de gaten houdt, een controleur die voor het onderzoek werkt en zo nodig de Inspectie Gezondheidszorg en Jeugd. Zij houden uw gegevens geheim. Als u de toestemmingsverklaring ondertekent, geeft u toestemming voor het verzamelen, bewaren en inzien van uw medische en persoonsgegevens. Eenmalig wordt er tevens informatie opgevraagd over de uitgifte van uw medicatie (biologic) bij uw apotheek. Uw gegevens worden gecodeerd en gepseudonimiseerd door een beperkt aantal onderzoekers die hiervoor toestemming hebben gekregen. Ook al het materiaal (bloed) krijgt een unieke code in een beveiligd bestand voordat het verder wordt geanalyseerd in laboratoria. Namen en patiëntnummers zijn hieruit gehaald door de geautoriseerde onderzoekers van onze afdeling.

Na afloop van de studie wordt uw materiaal overgebracht en bewaard in een prospectieve research biobank (BIODIP) waarvan de doelstellingen overeen komen met de doelstellingen van deze studie. Een biobank is een faciliteit waar menselijk lichaamsmateriaal (zoals bloed, urine, weefsel) samen met bijkomende gegevens die betrekking hebben tot dit materiaal, worden bewaard. De medisch beheerder van deze biobank is Prof. Dr. Jo Lambert (UZ Gent, +32 (0)9/332.22.87, Jo.Lambert@uzgent.be).
Uw bloed wat afgenomen is voor deze studie zal 20 jaar (pseudoniem) bewaard worden. Zonder uw toestemming wordt hier verder geen onderzoek mee gedaan. Indien u dit op het formulier aangeeft, zouden we wel contact op kunnen nemen als er nieuwe onderzoeksvragen zijn. We zullen u dan vragen of we uw materiaal opnieuw mogen gebruiken. U krijgt dan ook uitleg over de nieuwe studie en moet opnieuw een toestemmingsformulier tekenen. U blijft echter “eigenaar” van uw lichaamsmateriaal. Dat betekent dat u steeds kan eisen dat de biobank uw opgeslagen materiaal vernietigt. U moet hiervoor contact opnemen met uw behandelend arts, die er dan voor zorgt dat uw opgeslagen lichaamsmateriaal wordt vernietigd.

Als u in de dosisafbouw groep zit, zullen we de huisarts op de hoogte brengen van studie deelname. Als u in de controle groep zit, zullen we de huisarts op de reguliere manier op de hoogte houden zoals al gedaan werd tijdens uw huidige behandeling.

Wat is het algemene belang van het onderzoek?
We krijgen met deze studie inzicht of we een deel van de patiënten langdurig met een lagere dosis kunnen behandelen waarbij de psoriasis nog steeds rustig blijft. Dit zal ertoe leiden dat mensen in totaal minder medicatie gedurende hun leven gebruiken, dat hun immuunsysteem minder onderdrukt is waardoor mogelijk minder bijwerkingen optreden en de behandelingen minder duur zijn.

Wat zijn de financiële consequenties indien u besluit deel te nemen?
U krijgt geen vergoeding voor deelname aan het onderzoek. Omdat sommige patiënten elke 6 maanden normaliter door hun arts gezien worden, en nu dus elke 3 maanden gezien worden, is er besloten dat reiskosten voor de visite na 3, 9 en 15 maanden vergoed worden. Indien u een extra keer dient te komen voor studie-gerelateerde doeleinden, zal daarvoor ook een reiskostenvergoeding gegeven worden.

Bent u verzekerd wanneer u aan het onderzoek meedoet?
Deze studie is door de medisch-ethische commissie (regio Arnhem-Nijmegen) goedgekeurd. Voor iedereen die meedoet aan dit onderzoek is een verzekering afgesloten. De verzekering dekt schade als gevolg van het onderzoek. Dit geldt voor zulke schade die naar boven komt tijdens het onderzoek, of binnen vier jaar na het einde van het onderzoek. In de toegevoegde bijlage vind u meer informatie over deze verzekering.

Wie krijgt bericht van onze bevindingen?
U krijgt geen persoonlijk bericht over de uitkomsten van het onderzoek tenzij u hierom vraagt. De gegevens van de onderzochte patiëntengroepen worden gepubliceerd in vakbladen en kenbaar gemaakt aan patiëntenverenigingen in Nederland en België (Psoriasis Patiënten Nederland, Psoriasis Liga Vlaanderen, GIPSO Asbl).

Wat gebeurt er na de studie?
Als dosis afbouwen duidelijk succesvol is gebleken mag u in principe gewoon doorgaan met de lagere dosering. We zullen dan in de dagelijkse praktijk ook andere patiënten aanbieden de dosis te verlagen; als u in de controle groep zat tijdens deze studie, kunt u dan dus ook gaan afbouwen. Als het afbouwen van de dosis echter niet succesvol is gebleken zullen we patiënten adviseren de normale dosering te blijven gebruiken.

Bij voorbaat hartelijk dank voor uw medewerking namens het onderzoeksteam.

Prof. E. de Jong
Dermatoloog

Dr. J van den Reek
Arts-onderzoeker

T. van Gaalen
Research coördinator

Wanneer u vragen heeft over het onderzoek kunt u contact opnemen met de uitvoerend onderzoekers:

NAAM ZIEKENHUIS
NAAM LOK.HOOFDONDERZOEKER + FUNCTIE
Telefoon: XXX
E-mail: XXX (indien van toepassing)

NAAM LOK. RESEARCH MED. + FUNCTIE
Telefoon: XXX
E-mail: XXX (indien van toepassing)

Radboudumc Nijmegen
Dr. J. van den Reek, arts-onderzoeker Drs. L. van der Schoot, arts-onderzoeker
Telefoon: 024 818 73 59 (ma-do); 024 361 72 40 (vrij)
E-mail: benebio.derma@radboudumc.nl

Wanneer u vragen heeft over het onderzoek en u deze wilt stellen aan een arts die betrokken is bij het onderzoek, kunt u contact opnemen met XXX.

Bij spoedgevallen kunt u bellen met het algemene nummer XXX en vragen naar de dienstdoende dermatoloog.

Wanneer u **vragen heeft over het onderzoek** en u wilt deze stellen aan een **arts die *niet* betrokken** is bij dit onderzoek, kunt u contact opnemen met dr. Driessen (secretariaat Dermatologie, Radboudumc, tel 024-3613724).

Als u klachten heeft over het onderzoek, kunt u dit melden bij de onderzoeker. Desgewenst kunt u contact opnemen met de [CONTACTGEGEVENS PATIËNTENVOORLICHTING/KLACHTENOPVANG]

Voor vragen omtrent **gegevensbescherming** kunt u contact opnemen met de [CONTACTEGEVENS FUNCTIONARIS VOOR DE GEGEVNSBESCHERMING VAN HET ZIEKENHUIS]

Bijlagen:
Verzekeringsinformatie patiënten
Toestemmingsverklaring

Bijlage: Verzekering

Verzekeringstekst proefpersoneninformatie; datum goedkeuring METC vanaf 1 juli 2015
Voor de deelnemers aan dit onderzoek is door Radboudumc een verzekering afgesloten. Deze verzekering dekt schade door dood of letsel die het gevolg is van deelname aan het onderzoek, en die zich gedurende de deelname van de proefpersoon aan het onderzoek openbaart, of binnen vier jaar na beëindiging van diens deelname aan het onderzoek. De schade wordt geacht zich te hebben geopenbaard wanneer deze bij de verzekeraar is gemeld.

Bij schade kunt u direct contact leggen met de verzekeraar.

<u>De verzekeraar van het onderzoek is:</u>
Onderlinge Waarborgmaatschappij Centramed B.A.
Postbus 7374
2701 AJ Zoetermeer
Tel. 070 3017070
E-mail: Schade@centramed.nl

De verzekering biedt een maximum dekking van € 650.000,-- per proefpersoon en € 5.000.000,-- voor het gehele onderzoek en € 7.500.000,-- per jaar voor alle onderzoeken van dezelfde opdrachtgever. Bovenstaande bedragen zijn opgenomen in het Besluit verplichte verzekering bij medisch-wetenschappelijk onderzoek met mensen. Informatie hierover kunt u vinden op de website van de Centrale Commissie Mensgebonden Onderzoek: http://www.ccmo.nl

De verzekering dekt schade die het gevolg is van het medisch-wetenschappelijk onderzoek. De verzekering dekt **niet**:
- Schade waarvan op grond van de aard van het onderzoek zeker of nagenoeg zeker was dat deze zich zou voordoen;
- Schade aan de gezondheid die ook zou zijn ontstaan indien u niet aan het onderzoek had deelgenomen;
- Schade door het niet (volledig) opvolgen van aanwijzingen of instructies door de proefpersoon;
- Schade aan uw nakomeling(en), als gevolg van een nadelige inwerking van het onderzoek op u of uw nakomeling(en);
- Schade door een bestaande behandelmethode bij onderzoek naar bestaande behandelmethoden;
- Schade die een gevolg is van het optreden van een risico waarvoor u in de schriftelijke informatie bent gewaarschuwd, tenzij het risico zich in ernstiger mate voordoet dan was voorzien of het risico uiterst onwaarschijnlijk was.

Versie 01-02-2017/CRCN

Bijlage: <u>TOESTEMMINGSVERKLARING</u>*

Voor deelname aan het wetenschappelijk onderzoek **'Dosisreductie van IL-17 of IL-23 remmende biologics op geleide van ziekteactiviteit bij psoriasis patiënten met stabiele lage ziekteactiviteit - een gerandomiseerde pragmatische studie.'** versie 1.4, 01-07-2020

Ik ben naar tevredenheid over het onderzoek geïnformeerd. Ik heb de schriftelijke informatie goed gelezen. Ik ben in de gelegenheid gesteld om vragen over het onderzoek te stellen. Mijn vragen zijn naar tevredenheid beantwoord. Ik heb goed over deelname aan het onderzoek kunnen nadenken zolang ik dit wenste. Ik heb het recht mijn toestemming op ieder moment weer in te trekken zonder dat ik daarvoor een reden hoef op te geven.

Mijn studiegegevens kunnen gedeeld worden met overheidsinstellingen voor volksgezondheid in lidstaten van de Europese Unie die de beslissing over de vergoeding van medische behandelingen helpen onderbouwen. Een van deze instellingen is het KCE in België. Meer informatie in verband met de opdracht van het KCE vindt u onder www.kce.fgov.be. Deze overheidsinstellingen kunnen mijn studiegegevens enkel analyseren en gebruiken om de behandeling(en) die onderdeel is/zijn van deze studie (d.i. secukinumab (Cosentyx®), ixekizumab (Taltz®), brodalumab (Kyntheum®), guselkumab (Tremfya®), risankizumab (Skyrizi®), tildrakizumab (Ilumetri®)) al of niet te vergoeden in hun land. Deze analyses zullen hoe dan ook steeds gebeuren op basis van gepseudonimiseerde gegevens. Dit betekent dat de onderzoekers die deze analyses uitvoeren mijn identiteit niet kunnen achterhalen. Deze onderzoekers zijn overigens ook steeds gehouden aan hun professionele geheimhoudingsplicht.

☐ Ik stem toe met deelname aan het onderzoek.

Dit betekent tevens dat mijn data en lichaamseigen materiaal 20 jaar bewaard mogen blijven

Ook betekent dit dat mijn huisarts schriftelijk informatie ontvangt over deelname aan deze studie.

☐ U mag in de toekomst contact opnemen voor vervolgonderzoek.

Naam :

Geboortedatum :

Handtekening : **Datum:**

E-mailadres: *(voor het toesturen van de online vragenlijsten)*

Ondergetekende verklaart dat de hierboven genoemde persoon mondeling en schriftelijk over het boven-vermelde onderzoek geïnformeerd is. Hij/zij verklaart tevens dat een voortijdige beëindiging van de deelname door bovengenoemde persoon, van geen enkele invloed zal zijn op de zorg die hem of haar toekomt.

Naam :

Functie :

Handtekening : **Datum:**

PATIENTENINFORMATIE: 'Het afbouwen van biologics bij psoriasis patiënten met lage ziekte-activiteit

Officiële titel: Dose reduction of the new generation biologics (IL17 and IL23 inhibitors) in psoriasis: A pragmatic, multicentre, randomized, controlled, non-inferiority study - BeNeBio study

Geachte heer/mevrouw,
Wij vragen u vriendelijk om mee te doen aan een medisch-wetenschappelijk onderzoek bij patiënten die lijden aan psoriasis in opdracht van Radboudumc (Nederland). U beslist zelf of u wilt meedoen. Voordat u de beslissing neemt, is het belangrijk om meer te weten over het onderzoek. Lees deze informatiebrief rustig door. Bespreek het met partner, vrienden of familie. Hebt u na het lezen nog vragen, dan kunt u terecht bij de onderzoeker. Ook kunt u terecht bij een onafhankelijk persoon, die veel weet van het onderzoek. U vindt de namen en telefoonnummers bij de contactgegevens aan het einde van deze brief.

Waarom dit onderzoek?
De afdelingen Dermatologie van het Radboudumc (Nederland), Universitair Ziekenhuis (UZ) Gent (België) en omliggende streekziekenhuizen doen onderzoek naar de behandeling van psoriasis. U gebruikt al enige tijd een biologic (secukinumab (Cosentyx®), ixekizumab (Taltz®), brodalumab (Kyntheum®), guselkumab (Tremfya®), risankizumab (Skyrizi®), tildrakizumab (Ilumetri®)) en uw huid reageert hier goed op. Omdat er over andere biologics aanwijzingen zijn dat een lagere dosis net zo effectief kan zijn, willen wij nagaan of dit bij deze middelen ook zo is. Dit kan ertoe leiden dat sommige mensen langdurig met een lagere dosis kunnen worden behandeld terwijl de psoriasis rustig blijft. Wij willen onderzoeken of dit mogelijk tot minder bijwerkingen (op de lange termijn) leidt en of de kosten dalen. Omdat we niet willen dat de ziekte onnodig erger wordt, willen wij dit onderzoek doen op een manier waarin we patiënten goed kunnen begeleiden. Mensen die niet goed reageren op de lagere dosis, kunnen weer terug naar hun normale dosering. Er zullen in totaal 244 personen aan deze studie deelnemen, waarvan 98 in België. Dit onderzoek wordt in België gefinancierd door het KCE (Federaal kenniscentrum voor de gezondheidszorg). De lokale onderzoeksarts is XXX.

Wat vragen wij van u, en wat is er anders dan de huidige behandeling die u krijgt?
Wij willen u graag elke 3 maanden op de polikliniek controleren gedurende 1,5 jaar in het kader van deze studie. Er worden twee groepen gemaakt, en door loting zal bepaald worden in welke groep u terecht komt.

Er zijn 2 mogelijkheden: het kan zijn dat u in de zogenaamde '**controlegroep'** terecht komt. U kunt dan uw huidige behandeling gewoon doorzetten. Wel vragen wij u gedurende de studie voor, tijdens of na ieder bezoek online (of papieren) vragenlijsten in te vullen en 1 buisje bloed af te staan. Zoals u wellicht gewend bent scoren wij de ernst van de psoriasis tijdens elk bezoek.

Als u in de '**afbouwgroep'** geloot wordt, dan zal de dosis langzaam afgebouwd worden voor zover de ziekteactiviteit en uw levenskwaliteit dit toelaten. We zullen maximaal tot de halve dosering van uw huidige behandeling afbouwen (als u bijvoorbeeld guselkumab (Tremfya®) 1 spuit per 8 weken gebruikt, zullen we het maximaal verlagen naar guselkumab 1 spuit per 16 weken). Als de

psoriasis teveel toeneemt of uw kwaliteit van leven score slechter wordt, zal de oude dosering in overleg met u hervat worden. Wij vragen u voor, tijdens of na elke visite online (of papieren) vragenlijsten in te vullen en 1 extra buisje bloed af te staan, zoveel mogelijk op een moment dat u al geprikt wordt vanwege uw behandeling. Zoals u wellicht gewend bent scoren wij de ernst van de psoriasis tijdens elke visite. De visites kunnen iets langer duren, met name tijdens de eerste visite, waarin we alles omtrent de studie uitleggen.

U zal ook gevraagd worden een dagboek bij te houden waarin u gegevens betreffende de geïnjecteerde biologic, eventuele gezondheidsproblemen en nieuwe medicatiegebruik kan noteren.

Hieronder ziet u een schematische voorstelling van elke studievisite.

**Studievisites**	**Studiehandelingen**
Initiatievisite*	- Bespreking BeNeBio studie + ondertekenen toestemmingsformulier<br>- Uitleg studiedagboekje voor de patiënt<br>- Vragenlijsten in te vullen door patiënt<br>- Verzamelen en noteren van relevante klinische informatie*<br>- Klinische evaluatie (PASI score) door arts*<br>- Bloedafname door verpleegkundige of arts<br>- Indeling in de 'controlegroep' of 'afbouwgroep'
Maand 3<br>Maand 6*<br>Maand 9<br>Maand 12*<br>Maand 15<br>Maand 18*	- Bespreking studiedagboekje (anamnese* – nevenwerkingen* - bijkomstige medicatie*)<br>- Overlopen data van de injecties<br>- Vragenlijsten in te vullen door patiënt<br>- Klinische evaluatie (PASI score) door arts*<br>- Bloedafname door verpleegkundige of arts<br>- Beslissing tot houden huidige dosis, afbouw van de dosis of hernemen van de vorige dosis
*Opm.: Handelingen die in kader van uw standaard zorgtraject afgenomen worden, of m.a.w. die onafhankelijk van uw deelname aan deze studie worden uitgevoerd, worden in bovenstaande tabel weergegeven met een **	

Wat gebeurt er als u niet wilt deelnemen aan dit onderzoek?
Wij benadrukken dat uw deelname volledig vrijwillig is. Met andere woorden: u hoeft niet deel te nemen als u dit niet wilt en er verandert dan niets aan uw huidige behandeling. Daarnaast kunt u stoppen met de studie op ieder moment dat u dat wenst. Op dat moment zullen de reeds verzamelde gegevens in de databank blijven voor analyse, maar er zal geen nieuwe data toegevoegd worden.
Als u wenst deel te nemen aan dit onderzoek verzoeken wij u een schriftelijke toestemming te ondertekenen. Dit betekent dat u deze informatie heeft gelezen en begrepen. Tevens geeft u toestemming aan onderzoekers, aangesloten aan dit onderzoek, om in uw dossier gegevens t.a.v. de studie op te zoeken. De studiegegevens zullen gepseudonimiseerd worden (hierbij kan men uw

gegevens nog terug koppelen naar uw persoonlijk dossier) vastgelegd in een digitaal gegevensbestand; uw naam en geboortedatum worden niet opgeslagen.

Wat zijn de voor- en nadelen van deelname voor u, en welke bijwerkingen kunt u verwachten?
U hebt 67% kans dat u in de groep terecht komt waarin u uw medicatie het komende jaar gaat afbouwen. Als uw psoriasis rustig blijft, en u er een goede kwaliteit van leven bij behoudt, zal u op de halve dosering uitkomen van wat u nu gebruikt. Concreet dient u uzelf dus minder injecties toe. Mogelijk zien we dat u minder kans op (lange termijn) bijwerkingen krijgt, dit weten we nog niet en moet onderzocht worden. Soms komt het voor dat de psoriasis toeneemt. We zullen dan de oude dosering hervatten of u zonodig behandelen met extra medicatie. **U kunt tussen de visites door contact met ons opnemen zodat u niet hoeft te wachten tot u weer een afspraak heeft.** U hoeft daarna niet opnieuw af te bouwen maar we blijven u wel op de polikliniek controleren en vragenlijsten afnemen gedurende de studie. Patiënten die ook **'artritis psoriatica'** hebben, gewrichtsontstekingen passend bij psoriasis, kunnen mogelijk een toename van de gewrichtsklachten krijgen bij het minderen van de dosis. Wij zullen op voorhand ook met uw reumatoloog overleggen of hij/zij akkoord gaat met het afbouwen van uw dosis. Wij willen u ook vragen contact met uw behandelende dermatoloog en reumatoloog op te nemen wanneer dit het geval is. Mogelijk is er een kleine kans dat u antistoffen tegen de biologic gaat vormen en hierdoor de biologic minder goed gaat werken. Wij denken dat deze kans zeer klein of zelfs afwezig is. Wel willen we dit graag onderzoeken en prikken daarom extra bloed. Als u niet afbouwt, hebt u geen direct voordeel van dit onderzoek. Wel zult u in toekomst van de opgedane kennis kunnen profiteren omdat wij de resultaten in de praktijk willen toepassen.

Het onderzoek kost u elke 3 maanden tijd (ongeveer 20 minuten) om de vragenlijsten in te vullen, ongeacht in welke behandelgroep u terecht komt. Extra bloedbepalingen worden zoveel mogelijk tijdens uw normale bloedprikafspraken meegenomen, dus u hoeft in principe niet extra geprikt te worden tenzij u normaliter niet elke 3 maanden geprikt werd. Er wordt 1 extra buisje bloed afgenomen van 10 ml.

Hoe is de privacy geregeld bij dit onderzoek en wat gebeurt er met het lichaamsmateriaal?
In overeenstemming met de Belgische wet van 22 augustus 2002 betreffende de rechten van de patiënt, de Algemene Verordening Gegevensbescherming (of GDPR) (EU) 2016/679 van 27 april 2016 (die vanaf 25 mei 2018 in voege is) en de Belgische wet van 30 juli 2018, betreffende de bescherming van natuurlijke personen in verband met de verwerking van persoonsgegevens en betreffende het vrije verkeer van die gegevens, zal uw persoonlijke levenssfeer worden gerespecteerd en kan u toegang krijgen tot de verzamelde gegevens. Elk onjuist gegeven kan op uw verzoek verbeterd worden.
Uw toestemming om deel te nemen aan de studie betekent dat we gegevens van u verwerken voor het doel van de klinische studie. Deze verwerking van gegevens is wettelijk voorzien op basis van artikel 6, § 1, (b), (e) or (f) en artikel 9, § 2(j) van de Algemene Verordening Gegevensbescherming.

Mensen die uw gegevens kunnen inzien zijn het onderzoeksteam, vertegenwoordigers van de opdrachtgever, de veiligheidscommissie die het onderzoek in de gaten houdt, de Commissie voor Medische Ethiek, een controleur die voor het onderzoek werkt en zonodig de Inspectie voor de Gezondheidszorg en bevoegde overheden. Zij houden uw gegevens geheim. Als u de toestemmingsverklaring ondertekent, worden uw medische en persoonsgegevens verzameld,

bewaard en ingezien. In deze studie zullen ook gegevens verzameld worden via vragenlijsten. Daartoe zal u gevraagd worden een persoonlijk email-adres te bezorgen waarop u deze vragenlijst wenst te ontvangen.
Uw gegevens worden gepseudonimiseerd door een beperkt aantal geautoriseerde onderzoekers. Ook al het materiaal (bloed) krijgt een unieke code in een beveiligd bestand voordat het verder wordt geanalyseerd in laboratoria binnen België. Namen en patiëntnummers zijn hieruit gehaald door de geautoriseerde onderzoekers van onze afdeling.
De onderzoeker bewaart uw gegevens 25 jaar. De algemene verwerkingsverantwoordelijke van de gegevens is de internationale opdrachtgever Radboudumc in Nederland. In België is de verantwoordelijke het nationaal coördinerend centrum UZ Gent. Beide zijn verantwoordelijk voor de verwerking van uw gegevens. Echter, enkel het onderzoeksteam van de onderzoeker in uw ziekenhuis, XXX (aan te vullen met de naam van de hoofdonderzoeker in uw lokaal ziekenhuis), zal toegang krijgen tot uw persoonlijke gegevens. De Data Protection Officer kan u desgewenst meer informatie verschaffen over de bescherming van uw persoonsgegevens. Contactgegevens (gelieve de lokale contactgegevens aan te vullen):

Het belangrijkste doel van dit onderzoek is om de gezondheidszorg te verbeteren. Als de studie aantoont dat het verlagen van de dosis van een biologic even goed werkt als de standaarddosis, zullen de onderzoeksgegevens vervolgens worden gebruikt om na te gaan of de prijs-kwaliteitsverhouding ook beter is. Hiervoor kunnen uw gepseudonimiseerde gegevens gedeeld worden aan Belgische instanties zoals het KCE (federaal kenniscentrum voor de gezondheidszorg) en mogelijk andere federale instanties, RIZIV, of instanties binnen Europa met betrekking tot de gezondheidszorg. Dit o.a. voor verdere data analyse. Voor deze verdere analyses zal uw rijksregisternummer gevraagd worden en gebruikt worden door een vertrouwde derde partij (TTP, eHealth Platform), om uw studiegegevens te koppelen aan gegevens uit andere bronnen (facturatiegegevens voor zorg en minimale klinische gegevens verzameld tijdens een hospitalisatie). De onderzoekers die de aanvullende analyses uitvoeren zullen in geen geval uw identiteit zien, en alle onderzoekers zullen gehouden zijn aan hun professionele geheimhoudingsplicht.

Na afloop van de studie zullen uw stalen worden overgebracht en bewaard in een prospectieve research biobank (BIODIP) waarvan de doelstellingen overeen komen met de doelstellingen van deze studie. Een biobank is een faciliteit waar menselijk lichaamsmateriaal (zoals bloed, urine, weefselstalen…) samen met bijkomende gegevens die betrekking hebben tot dit materiaal, worden bewaard. De medisch beheerder van deze biobank is Prof. Dr. Jo Lambert (UZ Gent, +32 (0)9/332.22.87, Jo.Lambert@uzgent.be).
Uw bloed wat afgenomen is voor deze studie zal 20 jaar (pseudoniem) bewaard worden. Zonder uw toestemming wordt hier verder geen onderzoek mee gedaan. Indien u dit op het formulier aangeeft, zouden we wel contact op kunnen nemen als er nieuwe onderzoeksvragen zijn. We zullen u dan vragen of we uw materiaal opnieuw mogen gebruiken voor toekomstig onderzoek goedgekeurd door een erkend Belgisch ethisch comité. U krijgt dan ook uitleg over de nieuwe studie en moet opnieuw een toestemmingsformulier tekenen. Deelname aan toekomstig onderzoek is vrijblijvend. U blijft "eigenaar" van uw lichaamsmateriaal. Dat betekent dat u steeds kan eisen dat de biobank uw opgeslagen stalen vernietigt. U moet hiervoor contact opnemen met uw behandelende arts, die er dan voor zorgt dat uw opgeslagen lichaamsmateriaal wordt vernietigd.

Als u in de afbouwgroep zit, zullen we de huisarts op de hoogte brengen van uw deelname aan de studie. Als u in de controlegroep zit, zullen we de huisarts op de reguliere manier op de hoogte houden zoals al gedaan werd tijdens uw huidige behandeling.

Wordt u geïnformeerd als er tussentijds voor u relevante informatie over de studie bekend wordt?
U heeft te allen tijde het recht om vragen te stellen over de mogelijke en/of bekende risico's van deze studie. In de loop van het onderzoek worden gegevens onthuld die invloed kunnen hebben op uw bereidwilligheid om aan dit onderzoek deel te blijven nemen. Als u door uw deelname aan het onderzoek een nadeel ondervindt, krijgt u een passende behandeling.
Wat is het algemene belang van het onderzoek?
We krijgen met deze studie inzicht of we een deel van de patiënten langdurig met een lagere dosis kunnen behandelen waarbij de psoriasis nog steeds rustig blijft. Dit zal ertoe leiden dat mensen in totaal minder medicatie gedurende hun leven gebruiken, dat hun immuunsysteem minder onderdrukt is waardoor mogelijk minder bijwerkingen optreden en de behandelingen minder duur zijn.

Wat zijn de financiële consequenties indien u besluit deel te nemen?
U krijgt geen financiële vergoeding voor deelname aan het onderzoek. De extra consultaties in dienst van het onderzoek, buiten de standaardopvolging, zijn wel gratis (maand 3, 9, en 15). Ook worden eventuele transport- en parkeerkosten voor de extra studiebezoeken gecompenseerd door middel van een voucher ter waarde van 25€. U ondervindt geen extra kosten door deelname aan deze studie. Op het einde van de studie wordt het remgeld voor de studiemedicatie gecompenseerd door middel van een voucher.

Bent u verzekerd wanneer u aan het onderzoek meedoet?

Deze studie werd vooraf goedgekeurd door een onafhankelijke Commissie voor Medische Ethiek verbonden aan het Universitair Ziekenhuis van Gent en de Universiteit Gent na raadpleging van de ethische commissies van elk Belgisch centrum waar deze studie zal worden uitgevoerd. De studie wordt uitgevoerd volgens de richtlijnen voor de goede klinische praktijk (ICH/GCP) en de verklaring van Helsinki opgesteld ter bescherming van mensen deelnemend aan klinische studies. In geen geval dient u de goedkeuring door de Commissie voor Medische Ethiek te beschouwen als een aanzet tot deelname aan deze studie.

De opdrachtgever voorziet in een vergoeding en/of medische behandeling in het geval van schade en/of letsel ten gevolge van deelname aan deze klinische studie. Voor dit doeleinde heeft het Universitair Ziekenhuis Gent een verzekering afgesloten met foutloze aansprakelijkheid conform de wet inzake experimenten op de menselijke persoon van 7 mei 2004 (Allianz Global Corporate & Specialty – polisnummer BEL000862). Indien de arts-onderzoeker van mening is dat er verband met de studie mogelijk is (er is geen verband met de studie bij schade ten gevolge van het natuurlijke verloop van de ziekte of ten gevolge van gekende bijwerkingen van de standaardbehandeling), zal hij/zij de aangifteprocedure bij de verzekering starten. Op dat ogenblik kunnen uw gegevens doorgegeven worden aan de verzekeraar. In het geval van onenigheid met de arts-onderzoeker of met de door de verzekeringsmaatschappij aangestelde expert, en steeds wanneer u dit nodig acht, kunnen u, of in geval van overlijden uw rechthebbenden, de verzekeraar

rechtstreeks in België dagvaarden (Allianz Global Corporate & Specialty; Uitbreidingstraat 86, 2600 Berchem; Tel: +32 33 04 16 00).

Wie krijgt bericht van onze bevindingen?
U krijgt geen persoonlijk bericht over de uitkomsten van het onderzoek tenzij u hierom vraagt. De gegevens van de onderzochte patiëntengroepen worden gepubliceerd in vakbladen en kenbaar gemaakt aan patiëntenverenigingen in Nederland en België (Psoriasis Patiënten Nederland, Psoriasis Liga Vlaanderen, GIPSO Asbl).

Wat gebeurt er na de studie?
Als dosis afbouwen duidelijk succesvol is gebleken mag u in principe gewoon doorgaan met de lagere dosering. We zullen dan in de dagelijkse praktijk ook andere patiënten aanbieden de dosis te verlagen; als u in de controlegroep zat tijdens deze studie, kunt u dan dus ook gaan afbouwen. Als het afbouwen van de dosis echter niet succesvol is gebleken, zullen we patiënten adviseren de normale dosering te blijven gebruiken.

Bij voorbaat hartelijk dank voor uw medewerking.

Prof.	Lynda Grine
Nationaal Coördinator,<br>Dermatoloog UZ Gent	Research coördinator,<br>UZ Gent
Tel 09-3322287	Tel 09-3325117

Wanneer u vragen heeft over het onderzoek en u deze wilt stellen aan een arts die betrokken is bij het onderzoek kunt u contact opnemen met XXXXXX (contactgegevens).

In spoedgevallen kunt u bellen met het algemene nummer XXX en vragen naar de dienstdoende dermatoloog.

Als u klachten heeft over het onderzoek, kunt u dit melden bij de onderzoeker.

Als u vragen heeft over de bescherming van uw persoonsgegevens, kan de Data Protection Officer u hieromtrent meer informatie verschaffen.

Contactgegevens: XXX
Emailadres: XXX

De Belgische toezichthoudende instantie die verantwoordelijk is voor het handhaven van de wetgeving inzake gegevensbescherming is bereikbaar via onderstaande contactgegevens:

Gegevensbeschermingsautoriteit (GBA)
Drukpersstraat 35 – 1000 Brussel
Tel. +32 2 274 48 00
e-mail: contact@apd-gba.be

Website: www.gegevensbeschermingsautoriteit.be

Bijlagen:
Toestemmingsformulier

BeNeBio

Bijlage: <u>TOESTEMMINGSFORMULIER</u>

Voor deelname aan het wetenschappelijk onderzoek **'Dosisreductie van biologics (IL-17 of IL-23 remmers) op geleide van ziekteactiviteit bij psoriasis patiënten met stabiele lage ziekteactiviteit'**

'Dose reduction of the new generation biologics (IL17 and IL23 inhibitors) in psoriasis: A pragmatic, multicentre, randomized, controlled, non-inferiority study - BeNeBio study'

□ Ik ben naar tevredenheid over het onderzoek geïnformeerd. Ik heb de schriftelijke informatie goed gelezen en een kopij gekregen. Ik ben in de gelegenheid gesteld om vragen over het onderzoek te stellen. Mijn vragen zijn naar tevredenheid beantwoord. Ik heb goed over deelname aan het onderzoek kunnen nadenken zolang ik dit wenste. Ik heb het recht mijn toestemming op ieder moment weer in te trekken zonder dat ik daarvoor een reden hoef op te geven. Ik stem ermee in om volledig samen te werken met de toeziende arts. Ik zal hem/haar op de hoogte brengen als ik onverwachte of ongebruikelijke symptomen opmerk.

□ Ik begrijp dat het onderzoeksteam, vertegenwoordigers van de opdrachtgever, de veiligheidscommissie die het onderzoek in de gaten houdt, de Commissie voor Medische Ethiek, een controleur die voor het onderzoek werkt en zonodig de Inspectie voor de Gezondheidszorg en bevoegde overheden mijn gegevens mogelijks willen inspecteren om de verzamelde informatie te controleren. Door dit document te ondertekenen geef ik toestemming voor deze controle. Bovendien ben ik op de hoogte dat mijn studiegegevens kunnen gedeeld worden met overheidsinstellingen voor volksgezondheid in lidstaten van de Europese Unie die de beslissing over de vergoeding van medische behandelingen helpen onderbouwen. Een van deze instellingen is het KCE in België. Meer informatie in verband met de opdracht van het KCE vindt u onder www.kce.fgov.be. Deze overheidsinstellingen kunnen mijn studiegegevens enkel analyseren en gebruiken om de behandeling(en) die onderdeel is/zijn van deze studie (d.i. secukinumab (Cosentyx®), ixekizumab (Taltz®), brodalumab (Kyntheum®), guselkumab (Tremfya®), risankizumab (Skyrizi®), tildrakizumab (Ilumetri®)) al of niet te vergoeden in hun land. Deze analyses zullen hoe dan ook steeds gebeuren op basis van gepseudonimiseerde gegevens. Dit betekent dat de onderzoekers die deze analyses uitvoeren mijn identiteit niet kunnen achterhalen. Deze onderzoekers zijn overigens ook steeds gehouden aan hun professionele geheimhoudingsplicht.

□ Ik begrijp dat mijn huisarts wordt ingelicht over mijn deelname aan deze studie.

□ Men heeft mij ingelicht dat zowel persoonlijke gegevens als gegevens aangaande mijn gezondheid worden verwerkt en bewaard gedurende minstens 25 jaar. Ik ben op de hoogte dat ik recht heb op toegang en op verbetering van deze gegevens. Aangezien deze gegevens verwerkt worden in het kader van medisch-wetenschappelijke doeleinden, begrijp ik dat de toegang tot mijn gegevens kan uitgesteld worden tot na beëindiging van het onderzoek. Indien ik toegang wil tot mijn gegevens, zal ik mij richten tot de arts-onderzoeker die verantwoordelijk is voor de verwerking.

Ik stem toe om deel te nemen aan volgende delen van de studie:

□ Deelname aan afbouwschema van mijn biologic (indien in afbouwgroep)

□ Afname en analyse van bloedstalen en verdere bewaring in een biobank (BIODIP UZ Gent; medisch beheerder: Prof. Jo Lambert) voor een periode van 20 jaar

□ Afname vragenlijsten

□ Ik ga akkoord dat mijn e-mailadres gebruikt wordt voor het toesturen van de online vragenlijsten.
E-MAILADRES: ……….

□ Bijhouden dagboek

□ Ik stem ermee in dat in de toekomst op vraag van een wetenschappelijk tijdschrift mijn geanomiseerde gegevens publiekelijk gepubliceerd mogen worden.

□ Ik ga akkoord en aanvaard dat mijn gepseudonimiseerde gegevens en/of materiaal gedeeld en/of verwerkt kunnen worden met/door Belgische en Europese academische centra/instanties van Radboudumc en het UZ Gent, alsook het KCE en ziekte verzekeringsmaatschappijen (inclusief maar niet gelimiteerd tot het RIZIV), overheidsdiensten, instellingen of organen binnen de Europese Economische Ruimte.

□ Ik ga ermee akkoord dat mijn rijksregisternummer verzameld wordt door mijn behandelende arts en dat deze aan een betrouwbare derde partij gegeven kan worden. Deze betrouwbare derde partij kan vervolgens mijn rijksregister koppelen aan gegevens uit andere bronnen facturatiegegevens voor zorg en minimale klinische gegevens verzameld tijdens een hospitalisatie.□ Ik stem ermee in dat u mij in de toekomst kan contacteren voor vervolgonderzoek

□ Ik stem ermee in dat u mij in de toekomst kan contacteren voor vervolgonderzoek

□ **ALLEEN voor UZ Gent patiënten:** ik ga akkoord dat ik een tweetal dagen voor de geplande studievisite een sms of e-mail ontvang ter herinnering van de visite.

Naam en voornaam deelnemer :

Handtekening :

Datum:

Ondergetekende verklaart dat de hierboven genoemde persoon mondeling en schriftelijk over het bovenvermelde onderzoek geïnformeerd is en bevestigt dat geen enkele druk op de deelnemer is uitgeoefend om hem/haar te doen toestemmen tot deelname aan de studie. Hij/zij verklaart tevens dat een voortijdige beëindiging van de deelname door bovengenoemde persoon, van geen enkele invloed zal zijn op de zorg die hem of haar toekomt. Hij/zij bevestigt dat hij/zij werk in overeenstemming met de ethische beginselen zoals vermeld in de laatste versie van de "Verklaring

van Helsinki", de "Goede klinische praktijk" en de Belgische wet van 7 mei 2004 inzake experimenten op de menselijke persoon.

Naam en voornaam arts-onderzoeker :

Handtekening :

Datum:

Dit formulier is bestemd voor onderzoek met personen van 18 jaar en ouder die wilsbekwaam zijn. Bij dit soort onderzoek moet door de betrokkenen zelf toestemming worden verleend.

INFORMATIONS POUR LE PATIENT: 'Réduction de la dose d'agents biologiques agents biologiques (IL-17 ou IL-23 inhibiteurs) basée sur l'activité de la maladie chez des patients atteints de psoriasis présentant une activité stable de la maladie'

Titre officiel: Dose reduction of the new generation biologics (IL17 and IL23 inhibitors) in psoriasis: A pragmatic, multicentre, randomized, controlled, non-inferiority study - BeNeBio study

Cher(e) Madame/Monsieur,

Nous vous demandons de bien vouloir participer à une recherche médico-scientifique que nous menons chez des patients souffrant de psoriasis. Le promoteur de cette recherche est Radboudumc (Pays-Bas). Vous décidez si vous voulez participer. Avant de prendre votre décision, il est important d'en savoir plus sur la recherche. Lisez cette formulaire d'information à votre aise. Discutez-en avec votre partenaire, votre famille ou vos amis. Si vous avez encore des questions après d'avoir lu ce formulaire, vous pouvez contacter votre docteur. Vous pouvez également contacter une personne indépendante, qui pourrait vous informer sur la recherche. Vous trouverez les noms et numéros de téléphone avec les coordonnées à la fin de ce formulaire.

Pourquoi cette étude?

Les départements de dermatologie du Radboudumc (Pays-Bas), de l'Hôpital Universitaire de Gand (UZ Gent, Belgique) et des hôpitaux régionaux environnants mènent actuellement des recherches sur le traitement du psoriasis. Depuis quelque temps, vous utilisez un agent biologique (sécukinumab (Cosentyx®), ixekizumab (Taltz®), brodalumab (Kyntheum®), guselkumab (Tremfya®), risankizumab (Skyrizi®), tildrakizumab (Ilumetri®)) et votre peau réagit bien à cela. Sur base d'études réalisées sur d'autres agents biologiques, on sait qu'une dose plus faible pourrait être tout aussi efficace. Nous souhaitons vérifier si tel est également le cas chez les nouveaux agents biologiques. Ces tests peuvent amener certaines personnes à être traitées avec une dose plus faible pendant une période prolongée, durant laquelle le psoriasis restera stable. Nous pensons que cela aidera à réduire les effets secondaires à long terme et que les coûts diminueront. Parce que nous ne voulons pas que la maladie empire inutilement, nous voulons faire cette recherche de manière à pouvoir guider correctement les patients. Les personnes qui ne répondent pas bien à la dose réduite peuvent retourner à leur dose habituel.

Un total de 244 participants prendera part à cette recherche, dont 98 en Belgique. Cette recherche est financée en Belgique par le KCE (centre fédéral de connaissances pour la santé). Le médecin investigateur local est le XXX.

Qu'est-ce que nous vous demandons et qu'est-ce qui est différent du traitement actuel que vous recevez?

Nous aimerions vous contrôler tous les 3 mois à la clinique pendant un an et demi dans le cadre de cette étude. Deux groupes seront créés: vous êtes placé dans un groupe par tirage au sort.

Il y a 2 possibilités: vous pouvez vous retrouver dans le "groupe de contrôle". Vous pouvez alors continuer votre traitement actuel. Pendant l'étude, nous vous demandons cependant de remplir des questionnaires en ligne (ou sur papier) avant, pendant ou après chaque visite et de donner 1 tube de sang supplémentaire lors des injections régulières. Comme vous en avez peut-être l'habitude, nous évaluons la gravité du psoriasis à chaque visite.

Si vous êtes inclus dans le '**groupe de réduction de la dose**', la dose sera progressivement réduite dans la mesure de l'activité de la maladie et de votre qualité de vie. Nous réduirons au maximum jusqu'à la moitié de la dose de votre traitement actuel (par exemple, si vous utilisez 1 seringue de guselkumab (Tremfya®) toutes les 8 semaines, nous la passerons à 1 seringue de guselkumab toutes les 16 semaines). Si le psoriasis empire trop, ou que votre qualité de vie en souffre, l'ancienne dose sera reprise en consultation avec vous. Nous vous demanderons de remplir des questionnaires en ligne (ou sur papier) avant, pendant ou après chaque visite pendant un an et demi et de donner 1 tube de sang supplémentaire au moment où vous administrez vos injections d'agent biologique durant votre consultation habituelle. Comme vous le savez peut-être, nous évaluons la gravité du psoriasis à chaque visite. Les visites peuvent prendre un peu plus de temps, en particulier lors de la première visite, durant laquelle nous expliquons tout sur l'étude.

Nous aimerions vous demander de tenir un journal dans lequel vous pouvez consignez des données concernant les agents biologiques injectés, des problèmes de santé et l'utilisation de nouveaux médicaments.

Vous trouverez ci-dessous une représentation schématique de chaque visite d'étude.

**Visites de l'étude**	**Actions dans le cadre de l'étude**
Visite d'initiation*	- Discussion de l'étude BeNeBio + le formulaire de consentement à signer par le participant<br>- Expliquer le journal d'étude au participant<br>- Questionnaires à remplir par le participant<br>- Collection et enregistrement des informations cliniques pertinentes*<br>- Evaluation clinique (PASI) par le médecin*<br>- Prise de sang par une infirmière ou un médecin<br>- Classement dans le "groupe de contrôle" ou "groupe de réduction de la dose"<br>-
Mois 3<br>Mois 6*<br>Mois 9<br>Mois 12*<br>Mois 15<br>Mois 18*	- Discussion du journal d'étude (anamnèse* - effets secondaires* - médicaments supplémentaires*)<br>- Revue des dates des injections<br>- Questionnaires à remplir par le patient<br>- Evaluation clinique (PASI) par le médecin *<br>- Prise de sang par une infirmière ou un médecin<br>- Décision de conserver la dose actuelle, de réduire la dose ou de reprendre la dose précédente<br>-
*Remarque: Les actions prises dans le cadre de votre programme de soins standard, ou en d'autres termes, qui sont effectuées indépendamment de votre participation à cette étude, sont indiquées dans le tableau ci-dessus avec un **	

Que se passe-t-il si vous ne souhaitez pas participer à cette étude?

Nous soulignons que votre participation est totalement libre. En d'autres termes, vous n'êtes pas obligé de participer si vous ne le souhaitez pas et rien ne change à votre traitement actuel. Vous pouvez également arrêter l'étude à tout moment. Si vous êtes retiré de l'étude, les données codées déjà collectées resteront dans la base de données pour analyse, mais aucune nouvelle donnée ne sera ajoutée.
Si vous souhaitez participer, nous vous demandons de signer une autorisation écrite. Cela signifie que vous avez lu et compris ces informations. Vous autorisez également les chercheurs liés à cette étude, à rechercher des données relatives à l'étude dans votre dossier. Les données de l'étude seront enregistrées sous un code (de sorte que vos données peuvent encore être liées au dossier personnel) dans une base de données numériques; votre nom et date de naissance ne seront pas enregistrés.

Quels sont les avantages et les inconvénients de la participation pour vous et quels effets secondaires pouvez-vous attendre?

Vous avez 67% de chance de faire partie du groupe dans lequel vous réduirez vos médicaments au cours de la prochaine année. Si votre psoriasis reste calme et que vous conservez une bonne qualité de vie, vous vous retrouverez avec la moitié de la dose que vous utilisez actuellement. Concrètement, vous devriez vous faire moins d'injections. En plus, nous verrons peut-être que vous êtes moins susceptible de souffrir d'effets secondaires (à long terme), mais cela n'est pas connu et fera partie de l'étude. Parfois, le psoriasis empire. Nous reprendrons alors l'ancien dosage ou, si nécessaire, nous vous traiterons avec un médicament supplémentaire. **Vous pouvez nous contacter entre vos consultations afin de prendre un rendez-vous immédiat.** Vous n'êtes pas obligé de reprendre l'expérience après cela, mais nous continuerons à vous surveiller à la clinique externe et à vous soumettre des questionnaires pendant l'étude. Les patients souffrant également d'**arthrite psoriasique**, une inflammation articulaire liée au psoriasis, peuvent présenter une augmentation du nombre de problèmes articulaires lorsque la dose est réduite. Nous discuterons en avance avec votre rhumatologue pour savoir s'il/elle accepte la réduction de votre dose. Il y a peut-être un léger risque que vous formiez des anticorps contre l'agent biologique et par conséquent l'agent biologique fonctionnera moins bien. Nous considérons ce risque minime voir inexistent. Nous aimerions toutefois étudier cette possibilité et, par conséquent, collecter des échantillons de sang supplémentaire. Si vous êtes dans le groupe de contrôle, il n'y a pas des avantages immédiats pour vous. Cependant, à l'avenir, vous bénéficierez des résultats positifs de cette étude.
La recherche vous prendra tous les 3 mois environ 20 minutes afin de remplir les questionnaires, quel que soit le groupe de traitement dans lequel vous vous retrouvez. Des analyses de sang supplémentaires sont incluses autant que possible lors de votre prélèvement sanguin habituel. Si vous ne vous piquez pas tous les 3 mois en temps normal, un tube de sang supplémentaire de 10 ml sera prélevé dans ce cas.

Comment la vie privée est-elle réglementée dans cette étude et qu'advient-il du matériel corporel?
Conformément à la législation belge du 22 Août 2002 relative aux droits du patient, le Règlement général (UE) n°2016/679 relatif à la protection des données personnelles (ou RGPD) du 27 avril 2016 (en vigueur à compter du 25 mai 2018) et la loi belge du 30 juillet 2018, votre vie privée sera respectée et vous pouvez, si vous le souhaitez, avoir accès aux données collectées. Toute information incorrecte peut être corrigée à votre demande.

Votre consentement à participer à l'étude signifie que nous traitons vos données pour les besoins de l'étude clinique. Ce traitement de données est prévu par la loi sur la base de l'article 6, paragraphes 1, b), e) ou f) et de l'article 9, paragraphe 2 j) du Règlement général sur la protection des données.

Les personnes qui peuvent consulter vos données sont l'équipe de recherche, les représentants du promoteur de l'étude, le comité de sécurité qui surveille l'étude, le Comité d'éthique médicale, un superviseur travaillant pour l'étude et, si nécessaire, l'Inspection des Soins de Santé, et les autorités compétentes. Toutes personnes sont tenus à garder vos données secrètes. En signant la déclaration de consentement, vous acceptez que vos données médicales et personnelles sous forme codée seront collectées, stockées et inspectées. Dans cette étude, des données par questionnaires seront collectées aussi. Il vous sera demandé de fournir une adresse e-mail personnelle à laquelle les questionnaires seront envoyés.
Vos données codées sont collectées par un nombre limité de chercheurs autorisés. Même le matériel (le sang) reçoit un code unique dans un fichier sécurisé avant d'être analysé dans les laboratoires en Belgique. Les noms et les numéros de patients en ont été extraits par les chercheurs autorisés de notre département. Le chercheur gardera vos données pendant 25 ans et les échantillons de sang pendant 20 ans.
Le contrôleur des données est le promoteur international Radboudumc (Pays-Bas). En Belgique, le contrôleur est UZ Gent. Les deux sont responsables du traitement de vos données. Seule l'équipe de recherche de l'investigateur principal de l'étude, XXX aura accès à vos données personnelles dans le cadre de cette étude. Le Data Protection Officer peut vous fournir plus d'informations sur la protection de vos renseignements personnels. Vous pouvez contacter: XXX

Le but principal de cette étude est d'améliorer les soins de santé. Si l'étude montre que la réduction de la dose d'un agent biologique fonctionne aussi bien que la dose habituelle, les résultats seront utilisés pour savoir si son rapport qualité-prix est également plus intéressant. Vos données médicales peuvent être transmises sous forme codée à des autorités Belges telles que le KCE (centre fédéral d'expertise pour les soins de santé) et éventuellement d'autres agences fédérales, INAMI, ou institutions en Europe liées aux soins de santé. Ceci par exemple pour d'autres analyses des données. Pour ces analyses supplémentaires, votre numéro national belge vous sera demandé et sera utilisé par une tierce personne de confiance (TTP, eHealth Platform) pour faire un lien entre vos résultats dans cette étude et des données provenant d'autres sources (données de facturation de soins et résumés cliniques minimaux collectés pendant votre séjour à l'hôpital). En aucun cas, les chercheurs effectuant les analyses supplémentaires ne connaîtront votre identité ; de plus, tous les chercheurs sont liés par le secret professionnel.

À la fin du recherche, vos prélevements (le sang) seront transférés dans une biobanque de recherche prospective (BIODIP) pour des recherches scientifiques futures, dont les objectifs correspondent à ceux de la présente étude. Une biobanque est une installation dans laquelle des échantillons corporels humains (sang, urine, échantillons de tissus, etc.) sont stockés avec des données supplémentaires relatives à ces échantillons. Le responsable médical de cette biobanque est le Prof. Dr. Jo Lambert (UZ Gent, +32 (0)9/332.22.87, Jo.Lambert@uzgent.be).
Votre sang prélevé pour cette étude sera conservé pendant 20 ans (sous un code). Sans votre consentement, aucune autre recherche n'aura lieu. Si vous l'indiquez sur le formulaire, nous pourrons vous contacter si de nouvelles questions de recherche se posent. Nous vous

demanderons ensuite si nous pouvons réutiliser votre matériel pour des recherches futures approuvées par un comité d'éthique belge reconnu. Vous recevrez également une explication de la nouvelle étude et vous devrez signer à nouveau un formulaire de consentement. La participation à ces recherches futures est optionnelle. Cependant, vous restez le "propriétaire" de vos échantillons. Cela signifie que vous pouvez toujours demander à la biobanque de détruire vos échantillons stockés. Pour cela, vous devez contacter votre médecin traitant, qui s'assurera que vos échantillons stockés soient détruits.

Si vous êtes dans le groupe de réduction de la dose, nous informerons votre médecin généraliste de votre participation à l'étude. Si vous faites partie du groupe de contrôle, nous informerons le médecin généraliste de la manière habituelle, comme cela a été fait pendant votre traitement actuel.

Êtes-vous informé si des informations provisoires sur l'étude sont connues entre-temps?
Vous avez le droit à tout moment de poser des questions sur les risques potentiels et/ou connus de cette étude. Au cours de l'étude, des données susceptibles d'affecter votre volonté de continuer à participer à cette étude vous seront révélées. Si vous rencontrez un désavantage en raison de votre participation à l'étude, vous recevrez un traitement approprié.

Quel est l'intérêt général de la recherche?
Cette étude nous permet de savoir si nous pouvons traiter une partie des patients avec une dose plus faible à long terme, lorsque le psoriasis reste stable. Cela mènera les personnes à consommer moins de médicaments au cours de leur vie, leur système immunitaire sera moins réprimé, ce qui réduira les effets secondaires et le coût des traitements.

Quelles sont les conséquences financières si vous décidez de participer?
Vous ne recevrez aucune compensation pour votre participation à l'étude. Les consultations hors des visites standardisées en rapport avec l'étude seront gratuites (mois 3, 9 et 15). Les frais de transport et stationnement seront également indemnisés pour les visites supplémentaires liées à l'étude par un bon d'une valeur de 25€. À la fin de l'étude, les franchises pour les médicaments d'étude seront également remboursées par un bon.

Êtes-vous assuré lorsque vous participez à l'étude?
Cette étude a été évaluée par une commission d'éthique médicale indépendante de l'hôpital universitaire de Gand (UZ Gent) et l'université de Gand. Après consultation des comités d'éthique de chaque centre belge où cette étude sera réalisée, l'étude a reçu un avis favorable. L'étude est menée conformément aux directives des bonnes pratiques cliniques (ICH/GCP) et à la Déclaration d'Helsinki, rédigées pour protéger les personnes qui participent aux études cliniques. En aucun cas vous ne devez prendre l'avis favorable du comité d'éthique comme une incitation à participer à cette étude

Le promoteur de l'étude prévoit une compensation et/ou un traitement médical en cas de dommage et/ou dégât causé lors de votre participation à l'étude clinique. Conformément à la législation belge du 7 mai 2004, relative aux expérimentations sur la personne humaine, l'Hôpital Universitaire de Gand a souscrit un contrat d'assurance « sans fautes » couvrant cette responsabilité (Allianz Global Corporate & Specialty - numéro de police BEL000862). Si le médecin estime qu'un lien avec l'étude est possible (l'assurance ne couvre pas la progression naturelle de

la maladie ou les effets secondaires connus du traitement normal), il/elle initiera la procédure de déclaration auprès de la compagnie d'assurance. Dans ce cas, vos coordonnées peuvent être communiquées à l'assureur. En cas de désaccord soit avec le médecin de l'étude, soit avec l'expert nommé par la compagnie d'assurances, ou à tout moment que vous jugerez utile, vous ou – en cas de décès – vos ayants droit pouvez assigner l'assureur directement en Belgique (Allianz Global Corporate & Specialty; Uitbreidingstraat 86, 2600 Berchem; Tel: +32 33 04 16 00).

Qui sera informé de nos découvertes?
Vous ne recevrez pas de communication personnel sur les résultats de la recherche à moins que vous ne le demandiez. Les données des groupes de patients examinés sont publiées dans des revues professionnelles et communiquées à des associations de patients néerlandaises et belges (Patients atteints de psoriasis aux Pays-Bas, Psoriasis Liga Vlaanderen, GIPSO Asbl).

Que se passe-t-il après l'étude?
Si la réduction de la dose est clairement couronnée de succès, vous pouvez essentiellement continuer avec la dose la plus faible. Nous proposerons ensuite à d'autres patients en pratique quotidienne de réduire également la dose; ceci sera aussi valable pour le groupe de contrôle. Cependant, si la réduction de la dose n'a pas été couronnée de succès, nous conseillons aux patients de continuer à utiliser la dose normale.

Merci d'avance pour votre coopération.

Prof.	Lynda Grine
Coordinateur National, Dermatologue UZ Gent	Coordinateur de recherche, UZ Gent
Tel 09-3322287	Tel 09-3325117

Si vous avez des questions sur l'étude et que vous souhaitez parler à un médecin impliqué dans la recherche, veuillez contacter XXX

En cas d'urgence, vous pouvez appeler le numéro général XXX et demander le service de dermatologie.

Si vous avez des plaintes à propos de l'étude, vous pouvez le signaler au docteur.

Pour toute question concernant la protection des données, vous pouvez contacter le responsable de la protection des données: XXX

L'autorité de surveillance belge responsable de l'application de la législation sur la protection des données peut être contactée via les coordonnées suivantes:

Autorité de protection des données (APD)
Rue de la Presse 35 – 1000 Bruxelles
Tel: +32 2 274 48 00
E-mail: contact@apd-gba.be

Site web: www.autoriteprotectiondonnees.be

Pièces jointes:
Formulaire de consentement

Annexe: FORMULAIRE DE CONSENTEMENT

Pour participer à l'étude scientifique **'Réduction de la dose de agents biologiques (IL-17 ou IL-23 inhibiteurs) basée sur l'activité de la maladie chez des patients atteints de psoriasis présentant une état stable de la maladie'**

'Dose reduction of the new generation biologics (IL17 and IL23 inhibitors) in psoriasis: A pragmatic, multicentre, randomized, controlled, non-inferiority study - BeNeBio study'

□ Je suis satisfait(e) des informations reçues sur l'étude. J'ai lu les informations écrites attentivement et j'en ai reçu une copie. J'ai eu l'occasion de poser des questions sur la recherche. Les réponses apportées à mes questions étaient satisfaisantes. J'ai pu réfléchir à participer à l'étude aussi longtemps que je le souhaitais. J'ai le droit de retirer mon consentement à tout moment sans avoir à donner de raison. J'accepte de coopérer pleinement avec le médecin investigateur. Je l'informerai si je remarque des symptômes inattendus ou inhabituels chez moi.

□ Je comprends que l'équipe de recherche, les représentants du promoteur de l'étude, le comité de sécurité qui surveille l'étude, le Comité d'éthique médicale, un superviseur travaillant pour l'étude et, si nécessaire, l'Inspection des Soins de Santé, et les autorités compétentes peuvent vouloir inspecter mes données pour vérifier les informations collectées en cours d'étude. En signant ce document, j'autorise cette inspection. De plus, je suis conscient(e) que les données de cette étude peuvent être partagées avec les institutions de santé publique des États membres de l'Union européenne, qui aident à justifier la décision de remboursement des traitements médicaux. Une de ces institutions est le KCE en Belgique. Plus d'informations sur la mission du KCE sont disponibles sur www.kce.fgov.be. Ces institutions gouvernementales peuvent uniquement analyser les données de de cette étude et les utiliser pour le (s) traitement (s) faisant partie de cette étude (à savoir (secukinumab (Cosentyx®), ixekizumab (Taltz®), brodalumab (Kyntheum®), guselkumab (Tremfya®), risankizumab (Skyrizi®), tildrakizumab (Ilumetri®)), qu'elles soient remboursées ou non dans leur pays. Ces analyses seront toujours basées sur des données codées . Cela signifie que les chercheurs qui effectuent ces analyses ne peuvent pas trouver mon identité. Ces chercheurs sont également toujours tenus à leur obligation de secret professionnel.

□ Je comprends que mon médecin sera informé de ma participation à cette recherche

□ J'ai été informé que les données personnelles et les données relatives à ma santé sont traitées et stockées pendant au moins 25 ans. Je suis conscient(e) que j'ai le droit d'accéder à ces informations et à les corriger. Comme ces données sont traitées à des fins médico-scientifiques, je comprends que l'accès à mes données peut être différé jusqu'à la fin de l'étude. Si je veux accéder à mes données, je m'adresserai au médecin-investigateur responsable.

J'accepte de participer aux parties suivantes de l'étude:

□ Réduire la dose des médicaments biologiques au cours de la recherche (si inclus dans le groupe de réduction de la dose)

□ Collecter et analyser les tubes de sang et stockage dans une biobanque (BIODIP UZ Gent; responsable medical: Prof. Dr. Jo Lambert) pendant une période de 20 ans

□ Remplir des questionnaires

□ J'accepte que mon adresse e-mail personelle soit utilisée pour envoyer les questionnaires en ligne.

ADRESSE E-MAIL: ……

□ Tenir un journal

□ J'accepte qu'à l'avenir mes données anonymes puissent être publiées à la demande d'une revue scientifique.

□ J'accepte que mes données codées et/ou prélevements puissent être partagés et/ou traités avec/par des centres/organismes académiques belges et européens de Radboudumc et UZ Gent, ainsi que le KCE et les compagnies d'assurance maladie (y compris, mais sans s'y limiter, le NIHDI), les services gouvernementaux, les institutions ou les organismes de l'Espace économique européen.

□ J'accepte que mon numéro de registre national soit collecté par mon médecin traitant et qu'il puisse être communiqué à un tiers fiable. Ce tiers fiable peut ensuite relier mon registre national à des données provenant d'autres sources, des données de facturation pour les soins et des données cliniques minimales collectées pendant une hospitalisation.

□ Vous pouvez me contacter pour des recherches futures basées sur ces données.

□ **UNIQUEMENT pour les patients de l'UZ Gent**: j'accepte de recevoir un SMS ou un e-mail deux jours avant la visite d'étude prévue pour rappeler la visite.

Nom et prénom participant(e):

Signature :

Date:

Le soussigné déclare que la personne susmentionnée a été informée oralement et par écrit de l'enquête susmentionnée et confirme qu'aucune pression n'a été exercée sur le/la participant(e) pour lui permettre de participer à l'étude. Il/elle déclare également qu'une cessation prématurée de la participation de la personne susmentionnée n'aura aucune influence sur les soins qui lui

sont dus. Il/elle confirme qu'il/elle travaille conformément aux principes éthiques énoncés dans la dernière version de la "Déclaration d'Helsinki", des "Bonnes pratiques cliniques" et de la loi belge du 7 mai 2004 relative aux expériences sur la personne humaine.

Nom et prénom investigateur:

Signature :

Date:

** Ce formulaire concerne une recherche sur des personnes âgées de 18 ans et plus qui ont un testament. Dans ce type d'enquête, les parties elles-mêmes doivent donner leur autorisation.*
